# Coordination Geometry and Structure–Property Relationships in Alkaline-Earth β‑Diketonate Complexes with 2‑Methylimidazole

**DOI:** 10.1021/acsomega.5c12982

**Published:** 2026-05-06

**Authors:** José Manuel Bravo-Arredondo, Anayeli Carrasco-Ruiz, José Andrés Reyes-Avendaño, María Josefina Robles-Águila, Dulce Yolotzin Medina-Velázquez, Efrain Rubio Rosas

**Affiliations:** † Benemérita Universidad Autónoma de Puebla, Instituto de Ciencias, Centro de Investigación en Dispositivos Semiconductores, Edificio 105 C, Boulevard 14 Sur y Av. San Claudio, Col. San Manuel, Puebla, Puebla C. P. 72570, México; ‡ 27776 Universidad Autónoma de Tlaxcala, Facultad de Ciencias Básicas, ingeniería y Tecnología, De Apizaquito, 20 de Noviembre, Apizaco, Tlaxcala 90401, México; § Benemérita Universidad Autónoma de Puebla, Facultad de Ingeniería Química, Ciudad Universitaria, Av. San Claudio y 18 Sur, Col. Jardines de San Manuel, Puebla, Puebla C. P. 72570, México; ∥ Benemérita Universidad Autónoma de Puebla, Centro Universitario de Vinculación y Transferencia de Tecnología, Puebla C. P. 72570, México

## Abstract

A series of alkaline-earth β-diketonate complexes derived from 2-thenoyltrifluoroacetone (TTA) and 2-methylimidazole was synthesized to study the influence of the metal ion (Mg^2+^, Ca^2+^, Sr^2+^, Ba^2+^) on coordination geometry, lattice organization, and thermal behavior. Single-crystal X-ray diffraction reveals that Ca–TTA crystallizes as a seven-coordinate [Ca­(TTA)_3_(H_2_O)]^−^ unit with capped trigonal prismatic CaO_7_ geometry, whereas Sr– and Ba–TTA form eight-coordinate [M­(TTA)_4_]^2 –^ complexes with distorted square-prismatic MO_8_ environments. The systematic increase in average M–O bond lengths (Ca–O 2.38 Å; Ba–O 2.74 Å) and unit-cell volume reflects expansion of the coordination sphere with increasing ionic radius. In contrast, Mg–TTA yields only microcrystalline material unsuitable for full structural resolution; spectroscopic and powder diffraction data suggest formation of a magnesium β-diketonate framework but without definitive crystallographic assignment. Infrared spectroscopy confirms deprotonation of TTA and consistent bidentate coordination across the series. A pronounced O–H stretching band is observed for Mg– and Ca–TTA, consistent with coordinated water identified crystallographically for Ca–TTA, while N–H bands in the 3150–3100 cm^–1^ region confirm incorporation of protonated 2-methylimidazolium counterions in all complexes. Thermogravimetric analysis reveals metal-dependent decomposition behavior, where Ca– and Sr–TTA exhibit staged degradation between 180 and 300 °C, Ba–TTA displays more clearly resolved stepwise decomposition at elevated temperatures, and Mg–TTA undergoes substantial early mass loss below 110 °C, indicating the removal of weakly bound, labile components or occluded species. Final residues (23–26%) are consistent with formation of metal-rich inorganic phases, likely oxides and/or carbonates. These results demonstrate how variation in alkaline-earth ionic radius modulates coordination number, lattice stabilization, crystallinity, and thermal response in TTA-based complexes.

## Graphical abstract

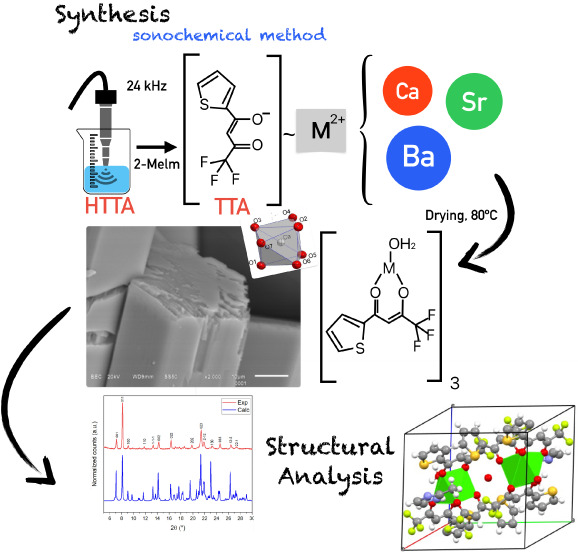

## 1 Introduction

Alkaline earth (Ae) metals, specifically Mg, Ca, Sr, and Ba, are attractive in coordination and materials chemistry due to their 2+ oxidation state, abundance, nucleophilic character, and size-dependent properties rather than merely serving as derivatives of Grignard reagents.^1–3^ These metals can accommodate high coordination numbers and diverse geometrical environments, which are directly related to reactivity and stability, and are beneficial for the development of functional materials such as catalysts, thin materials, and optical devices.^3–7^

Ae complexes are of significant interest because the increase in ionic radius and polarizability down Group 2 enables diverse coordination numbers and geometries, which influence structural and functional properties.^8–11^ These metals show technological potential in lightweight alloys, electronic devices, and biomedical systems, where Mg-based materials exhibit biodegradability and mechanical compatibility with bone tissue.^12–14^ Interest in Ae compounds has grown as environmentally friendly alternatives for advanced applications.^15,16^

Ae complexes with β-diketones and related donor ligands form an important yet incompletely understood class of coordination compounds at the interface of basic and applied inorganic chemistry.^17,18^ Since the pioneering crystallographic studies of magnesium and calcium β-diketonates, which laid the foundations of their basic coordination geometries and bonding characteristics.^19,20^ Subsequent work examined heteroleptic complexes and substituent effects on coordination and stability, highlighting their value as volatile and thermally stable precursors for chemical vapor deposition, thin-film growth, and supramolecular frameworks.^21–24^ Quantitative analyses of coordination numbers, bond lengths, and thermal behavior highlight their technological importance in catalysis and materials processing.^25,26^ However, the influence of β-diketone substituents and ancillary ligands on coordination geometry and metal–ligand bonding remains unclear. Although trends in aggregation and coordination number have been observed, steric and electronic effects are not yet predictive of structure–property relationships.^27^

Sterically bulky or multidentate ligands are employed to saturate Ae metal centers and suppress aggregation, a particular problem for these metals.^28–30^ In general, heavier congeners, such as Sr and Ba, are found to accommodate higher coordination numbers and more flexible geometries and thus show greater reactivity and broader utility than their Mg and Ca counterparts.^29,31^ Although beryllium formally belongs to Group 2, its β-diketonate chemistry is dominated by strongly covalent, low-coordination environments, tetrahedral coordination, and therefore differs fundamentally from the high-coordination ionic behavior characteristic of Mg–Ba complexes.^23^

2-Thenoyltrifluoroacetone (TTA) is a well-established β-diketonate ligand that promotes efficient ligand-to-metal energy transfer and enhances luminescence.^32–34^ Since its early application in europium-based systems, TTA is known for generating strong antenna effects that boost Eu^3+^ emission intensity and photostability, making it a benchmark for optoelectronic and photonic applications.^35,36^ The resulting TTA–Eu^3+^ complexes exhibit high photoluminescence quantum yields, up to 80%, and high thermal and chemical stability required for OLEDs, fluorescent coatings, and sensing platforms.^37^ However, the relationship between TTA molecular structure, crystal packing, and luminescent efficiency under varying conditions remains unresolved.^38–42^ There is also ongoing debate about divergent mechanistic explanations for the antenna effect, particularly regarding energy-transfer pathways and the structure-induced spectral tuning.^43^

Although Ae and related β-diketonate complexes, specifically with TTA, are promising compounds for optoelectronic, catalytic, and thin-film applications, the structural diversity and physicochemical tunability of these complexes across the Mg–Ba series have been insufficiently explored.^23,44^ Initial studies of Mg and Ca β-diketonates provided foundational understanding of their coordination modes and volatility, which have been further extended to more complex strontium and barium systems with higher nuclearities and bridging architectures.^20,21,45,46^ These complexes are key molecular precursors in metal–organic chemical vapor deposition (MOCVD) for superconducting and ferroelectric thin films, where volatility and thermal stability determine the efficiency and performance of the deposition.^47,48^ Despite decades of research, the relationship between coordination geometry, crystal packing, and metal–ligand interactions in these compounds and their impact on structural and thermal properties are not fully understood.^18,49^ While Mg complexes exhibit distorted octahedral coordination geometries,^45,50^ the heavier congeners exhibit a range of coordination numbers and aggregation tendencies, including monomeric and polymeric motifs with intricate μ-bridging.^51,52^

However, complexes of TTA with Ae ions (Ae–TTA) have not been extensively explored. The literature contains few examples of these complexes,^53,54^ despite the potential for these compounds to yield new and engaging properties in terms of spectroscopic, structural, and thermal behavior, which may include novel luminescent materials or metal–organic precursors with unique volatility profiles.^55^ Based on this, we report the synthesis and characterization of Mg, Ca, Sr, and Ba TTA complexes, focusing on structural, spectroscopic, and thermal trends across the series in relation to ionic radius and coordination preferences. This study expands understanding of Ae−β-diketonate chemistry and evaluates the potential of these complexes as thin-film precursors and functional materials.

## 2 Materials and Methods

### 2.1 Chemicals

All reagents used in these experiments were purchased from commercial sources at analytical pure grade and used without further purification. 2-Thenoyltrifluoroacetone (TTA, C_8_H_5_F_3_O_2_S, 99%), magnesium nitrate hexahydrate (Mg­(NO_3_)_2_·6H_2_O, > 99%), calcium nitrate tetrahydrate (Ca­(NO_3_)_2_·4H_2_O, > 99%), strontium nitrate anhydrous (Sr­(NO_3_)_2_, > 99%), barium triflate Ba­(CF_3_SO_3_)_2_, >98%, 2-methylimidazole (2-MeIm, C_4_H_6_N_2_, 99%), and methanol (CH_3_OH, > 99.8%) were purchased from Sigma-Aldrich. Deionized water was used for all experiments that were conducted.

### 2.2 Synthesis

Crystals of [2-MeImH]_m_[M^2+^(TTA)_n_] (M^2+^ = Mg^2+^, Ca^2+^, Sr^2+^, and Ba^2+^) were synthesized via a sonochemical method using a M^2+^:TTA:2-MeIm molar ratio of 1:2:1, as reported previously.^42^ The overall synthetic procedure is illustrated in Scheme 1. For each metal salt: Mg­(NO_3_)_2_·6H_2_O (0.591, 1 mmol), Ca­(NO_3_)_2_·4H_2_O (0.321, 1 mmol), Sr­(NO_3_)_2_ (0.225, 1 mmol), and Ba­(CF_3_SO_3_)_2_ (0.432 g, 1 mmol) were separately dissolved in 25 mL methanol. A ligand solution containing TTA (0.555 g, 2 mmol) and 2-methylimidazole (0.082 g, 1 mmol) was prepared in 25 mL of a 1:1 (v/v) methanol/deionized water mixture. The metal and ligand solutions were combined under stirring and subjected to sonication using an Ultrasonic Processor UP400St (24 kHz, 400 W, pulse mode: 1 pulse per second) for 20 min at room temperature, until precipitation occurred. The resulting suspensions were allowed to stand at room temperature for 2–4 h. The precipitates were isolated by centrifugation, washed with deionized water, and dried at ambient conditions overnight, affording single crystals suitable for X-ray diffraction. The isolated yields of the Ae–TTA complexes were consistently high and reproducible, ranging from approximately 86–93% depending on the Ae metal (Mg ≈93%, Ca ≈90%, Sr ≈88%, Ba ≈86%), based on the mass of the dried solid relative to the limiting metal precursor. In the case of Mg–TTA, only microcrystalline needles were obtained under identical conditions despite multiple recrystallization attempts; therefore single crystals suitable for SCXRD were unsuccessful to be isolated.

**Scheme 1:**
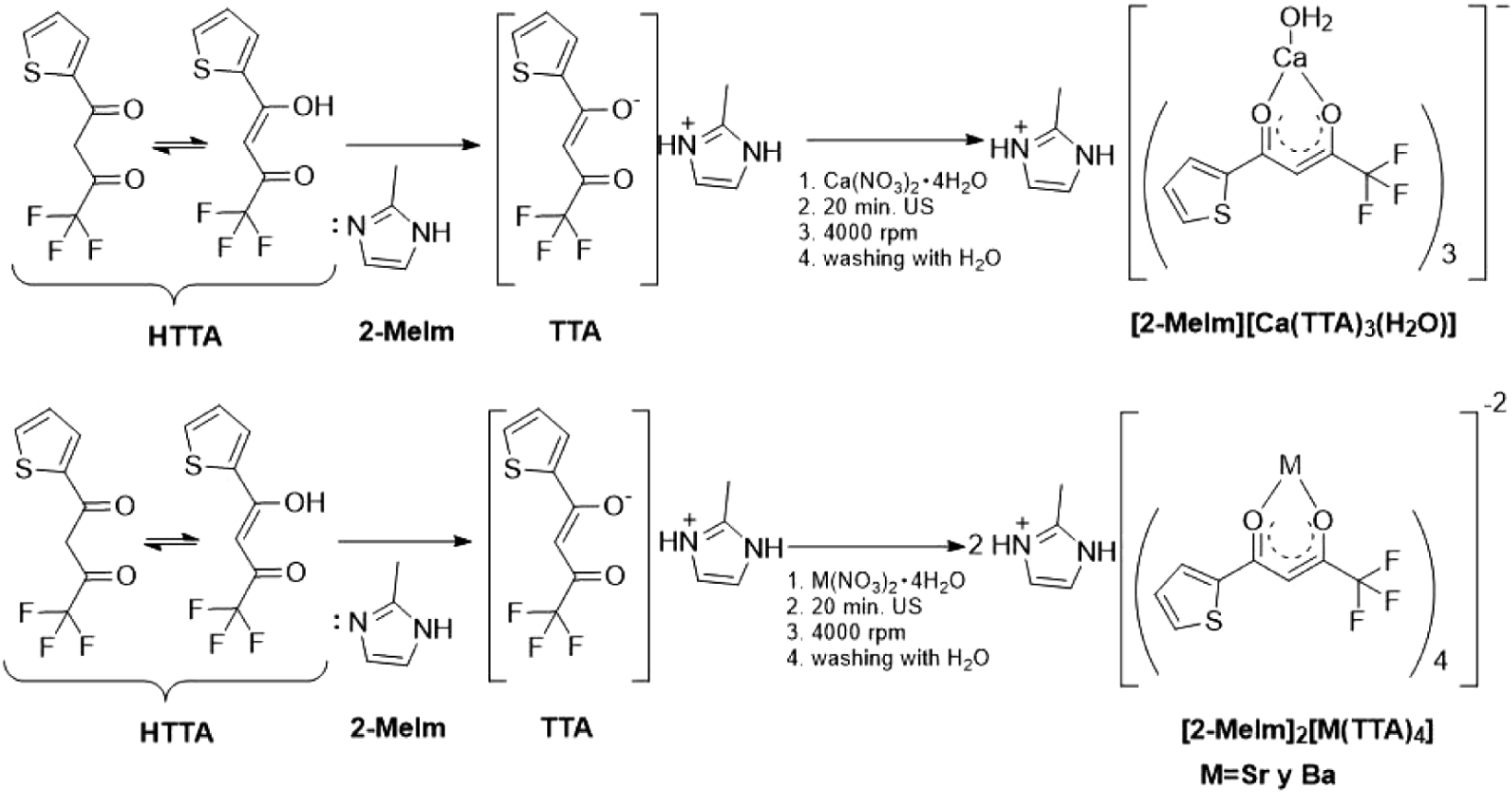
General Procedure for the Synthesis of Ae–TTA Complexes

### 2.3 Characterization Techniques

X-ray diffraction data were collected at 296 K on a Bruker D8 VENTURE diffractometer fitted with a CPAD-based detector using MoK\α radiation (λ = 0.71073 Å). The structure was solved by direct methods using Shelx-2018.^56^ Least-squares refinement based on F2 was carried out employing the full-matrix method of SHELXL-2018. All non-hydrogen atoms were refined with anisotropic thermal parameters. Hydrogen atoms were placed in calculated positions and refined with an isotropic fixed thermal parameter using a riding model. Molecular structure drawings were generated using Diamond 5.0 for Windows.^57^ Powder X-ray diffraction data were analyzed on a Bruker D8 Eco Advance fitted with a LynxEye detector, Bragg-Bretano goniometer geometry, a secondary graphite monochromator, and a copper anode (1.5456 Å). X-ray generator of 40 kV and 25 mA. The functional groups of the ligands (TTA and 2-MeIM) and Ae–TTA complexes were identified using an Attenuated Total Reflection of Fourier Transformed Infrared (ATR-FTIR) spectrometer within the 4000–500 cm^–1^ range recorded by PerkinElmer Spectrum One. Simultaneous thermal analysis was done on SDT Q600, TA Insruments, under nitrogen from room temperature to 550 °C with a ramp rate of 10 °/min. A JEOL JSM-6610LV model microscope was used, equipped with a tungsten filament, in a high-vacuum environment with a potential of 20 kV for the SEM analysis. The samples were mounted on carbon tape and coated with gold in a scan time of 60 s and × 500 and × 2000 amplification. All the measurements were performed at room temperature.

## 3 Results and Discussion

### 3.1 Single-Crystal X-ray Diffraction Analysis

The Single-Crystal X-ray Diffraction (SCXRD) technique was used to elucidate the solid-state structure. The molecular structures are shown in Figure 1a–c, and the crystal data collection and refinement details are given in Table 1. The solid-state structure of [C_3_H_6_N_2_]_2_[Ca­(TTA)_3_(H_2_O)], named as Ca–TTA complex, crystallizes in the triclinic P-1 space group and shows a capped trigonal prismatic molecular geometry around the central ion Ca^2+^ with a coordination number seven Figure 2a. It is defined by six oxygen atoms of the TTA ligands and one oxygen atom of the coordinated water. The average Ca–O bond length for TTA ligands is 2.378(5) Å, and the Ca–O bond length for coordinated water is 2.434(2) Å, consistent with the values reported for similar structures.^58^

**Figure 1:**
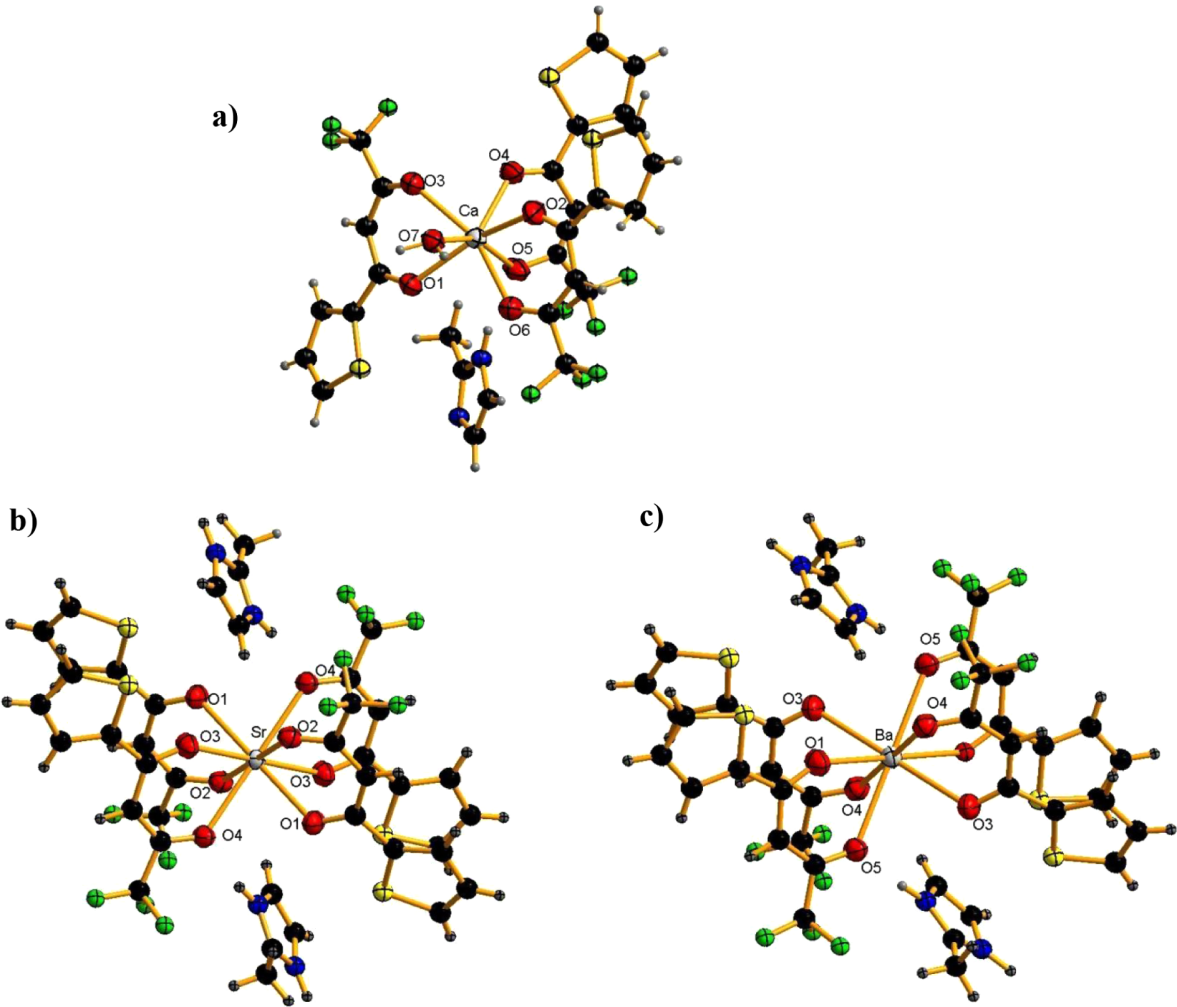
Crystal structure of **a)** Ca–TTA, **b)** Sr–TTA, and **c)** Ba–TTA.

**Figure 2:**
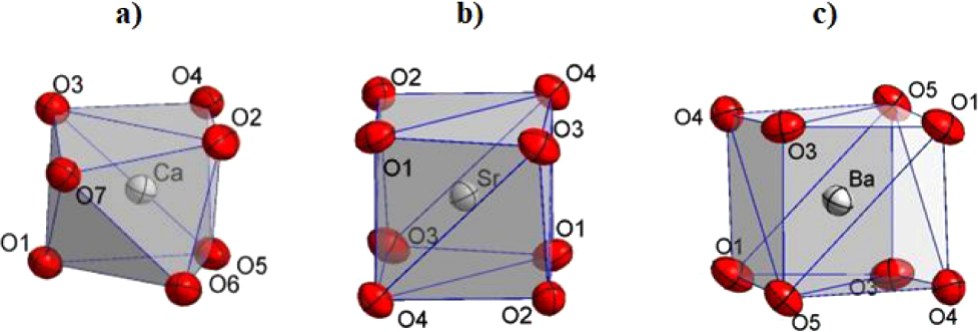
Local coordination environment of **a)** Ca–TTA, **b)** Sr-TTA, and **c)** Ba–TTA.

**Table 1:** Crystal Data and Structures Refinement of M-TTA Complexes

Formula	C_28_ H_20_ Ca F_9_ N_2_ O_7_ S_3_	C_40_ H_30_ F_12_ N_4_ O_8_ S_4_ Sr	C_40_ H_30_ F_12_ N_4_ O_8_ S_4_ Ba
MW (g/mol)	803.72	1138.54	1188.26
Temperature (K)	296 (2)	295 (2)	297 (2)
Crystal system, space group	Triclinic, P-1	Monoclinic, P 2_1_/c	Monoclinic, P 2_1_/c
*a* (Å)	10.2136 (5)	12.9496 (9)	13.2435 (4)
*b* (Å)	13.6443 (7)	14.9981 (10)	14.9433 (5)
*c* (Å)	13.7762 (7)	13.2216 (9)	13.1479 (4)
*α (°)*	71.499 (2)	90	90
*β (°)*	73.323 (2)	112.546 (3)	112.5270 (10)
*γ (°)*	87.744 (2)	90	90
V (Å^3^)	1741.19 (15)	2371.6 (3)	2403.46 (13)
Z	2	2	2
Density (g/cm^3^)	1.533	1.594	1.642
Absorption coefficient (mm^–1^)	0.456	1.414	1.100
F(000)	814	1144	1180
Crystal size	0.380 × 0.350 × 0.210 mm	0.430 × 0.340 × 0.080 mm	0.290 × 0.210 × 0.100 mm
Limiting indices	–13 ≤ *h* ≤ 14, −18 ≤ *k* ≤ 18, −18 ≤ *l* ≤ 19	–15 ≤ *h* ≤ 15, −17 ≤ *k* ≤ 17, −15 ≤ *l* ≤ 15	–17 ≤ *h* ≤ 17, −19 ≤ *k* ≤ 19, −17 ≤ *l* ≤ 15
*GOF on F2*	1.035	1.093	1.088
*Final R indices [I > 2sigma(I)]*	R_1_ = 0.0766, wR_2_ = 0.2281	R_1_ = 0.0496, wR_2_ = 0.1298	R_1_ = 0.0407, wR_2_ = 0.1015
*R indices (all data)*	R_1_ = 0.1062, wR_2_ = 0.2591	R_1_ = 0.0760, wR_2_ = 0.1590	R_1_ = 0.0712, wR_2_ = 0.1215
*Largest diff. peak and hole*	1.072 and −0.974 e.Å^–3^	0.806 and −0.654 e.Å^–3^	0.818 and −0.589 e.Å^–3^

On the other hand, the other two β-diketonate complexes crystallize in the monoclinic space group *P*2_1_/*c* with very similar unit-cell metrics, where [C_3_H_6_N_2_]_2_ [Sr­(TTA)_4_], and [C_3_H_6_N_2_]_2_ [Ba­(TTA)_4_], named as Sr–TTA and Ba–TTA complexes, respectively, show a square-based prismatic molecular geometry around the central metal ion with a coordination number eight Figure 2b,c. They are defined by eight oxygen atoms of TTA ligands, and the average Sr–O and Ba–O bonds for TTA ligands are 2.596(3) Å and 2.742(3) Å, respectively, as expected because of both their electronegativities and ionic radii.

In the Sr–TTA and Ba–TTA structures, the metal center lies on a crystallographic inversion center; consequently, each crystallographically independent M–O bond has an inversion-generated counterpart (O#i), and the eight oxygen donors are arranged as four trans O–M–O pairs with strictly linear angles (e.g., O(1)–M–O(#1) = 180°). The resulting MO8 coordination environment is therefore best described as a distorted square prism. The chelating bite of the TTA ligands leads to significant angular distortions, yielding complementary acute/obtuse angles such as O(1)–Ba(1)–O(3) = 73.79(9)° and O(1)–Ba(1)–O(#3) = 106.21(9)° (For Sr: O(1)–Sr(1)–O(3) = 71.69(10)° and O(1)–Sr(1)–O(#3) = 108.31(10)°). Selected bond lengths and angles are compiled in Tables S1–S2.

One notable difference is the presence of N–H hydrogen atoms for the Sr–TTA complex: the refined structure exhibits N(1)–H = 0.89 Å and N(2)–H = 0.86 Å, whereas no such N–H bonds were found in the Ba–TTA structure likely attributable to crystal packing effects, hydrogen-bond network and the degree of proton transfer stabilization, which primarily stems from the difference in ionic radii between Sr and Ba. These N–H moieties in the Sr–TTA complex could potentially participate in weak N–H···O interactions with nearby O atoms, although no short classical hydrogen bonds are present. Otherwise, van der Waals forces and π–π stacking between aromatic ligands are responsible for the crystal packing, and only weak C–H···O contacts are observed.

The crystallographic parameters of Ae–TTA complexes (Table 1) illustrate a trend in the cell volume, since Ba–TTA is larger (V = 2403.46(13) Å^3^) than for Sr–TTA (V = 2371.6(3) Å^3^) and for Ca–TTA (V = 1741.19(15) Å^3^). Also, the calculated density is correspondingly higher for Ba–TTA (1.642 g·cm^–3^) than for Sr–TTA (1.594 g·cm^–3^) and for Ca–TTA (1.533 g·cm^–3^). The other difference is that the asymmetric unit for Ba and Sr complexes consists of one metal center coordinated by four doubly deprotonated TTA ligands and two 2-MeImH. In contrast, for the Ca–TTA complex, the asymmetric unit has only three deprotonated TTA ligands, one 2-MeImH and one water molecule (Figure 1).

Within the unit cell of Ae–TTA complexes, the 2-methyl-4H-imidazol-3-ium (2-MeImH^+^) molecules serve as counterions. They are essential to the luminescent properties and the stability of the complex by forming the neutral ion-pair.^59^ In the solid state, the imidazolium is also a crucial component to the growth of the structure for Ca–TTA, with the + N⇀H moiety forming noncovalent charge-assisted hydrogen bond interactions between + N⇀H···O of + N···O distance 2.811(4) Å and C–H···O with the C⇀H moiety from imidazolium and C···O distances of 3.284(5) Å from the coordinated water molecule at the complex along the crystalline packing (Figure S1). The protonated imidazolium cations stabilized the charge imbalance generated by the TTA ligands, enabling more ordered packing and extended growth in the heavier Ae–TTA complexes.

To contextualize the coordination environments observed in the present Ca–, Sr–, and Ba–TTA complexes, a comparative analysis with representative Ae β-diketonate systems reported in the literature was performed (Table 2). This comparison highlights clear and systematic trends in coordination number, metal–oxygen bond distances, and angular distortion as a function of the Ae metal ionic radius.^60–65^

**Table 2:** Comparative Coordination Geometry Parameters for Ae β-Diketonate Complexes (M = Metal, CN = Coordination Number)

**M**	**Complex**	**Ligand Type**	**CN**	**Observed Geometry**	**M–O bond (Å)**	**O–M–O Bond Angle (°)**	**refs**
**Ca**	**[Ca(TTA)**_**3**_ **(H**_**2**_ **O)]**	**TTA**	**7**	**Capped Trigonal prism**	**2.38**	**74–157**	**This work**
	[Ca(acac)_2_(H_2_O)]·H_2_O	acac	7	Distorted capped octahedral	2.33	73–156	^60^
	[Ca(tmhd)_2_(tri)]	tmhd	7	Distorted capped trigonal prism	2.36	72–154	^61^
**Sr**	**[Sr(TTA)**_**4**_ **]**	**TTA**	**8**	**Square prism**	**2.60**	**68–150**	**This work**
	[Sr(dppd)_2_(tet)]	dppd	8	Distorted square prism	2.54	65–146	^62^
	[Sr(tmhd)_2_(bipy)]_2_	tmhd	8	Distorted square prism	2.56	66–147	^63^
**Ba**	**[Ba(TTA)**_**4**_ **]**	**TTA**	**8**	**Square prism**	**2.74**	**65–148**	**This work**
	[Ba(tmhd)_2_(bipy)_2_]_2_	tmhd	8	Expanded square prism	2.77	63–145	^64^
	[Ba(tmhd)_2_(NH_3_)_2_]_2_	tmhd	8	Expanded square prism	2.74	64–146	^65^

The observed geometrical evolution reflects the combined influence of ionic radius expansion, ligand denticity constraints, and lattice stabilization effects, consistent with established trends in Ae coordination chemistry. Calcium complexes typically adopt seven-coordinate environments, as exemplified by [Ca­(TTA)_3_(H_2_O)] and related acac (acetylacetonate) and tmhd (2,2,6,6-tetramethyl-3,5-heptanedione) ligands, with average Ca–O bond lengths of 2.33–2.38 Å and wide O–Ca–O bite-angle ranges of 72–157°, consistent with capped trigonal prismatic or closely related distorted polyhedra. Upon moving to strontium, an expansion to eight-coordinate environments is observed, with predominantly square prismatic geometries and longer average Sr–O distances (2.54–2.60 Å), using ligands as dppd (1,3-diphenylpropanedione) and tmhd, reflecting the larger ionic radius of Sr^2+^. For barium, the β-diketonate complexes remain eight-coordinate and square prismatic but exhibit further elongation of the metal–oxygen bonds (2.74–2.77 Å) together with a slightly narrower O–Ba–O angular distribution compared to Sr, indicating geometric expansion dominated by bond-length effects rather than increased angular distortion, even using tmhd ligand as well. Despite being isostructural with their Sr analogues, Ba complexes therefore display enhanced polyhedral size without a corresponding increase in angular flexibility.^60–65^

Overall, the comparative data in Table 2 demonstrate that the coordination geometries and metric parameters of the present Ca–, Sr–, and Ba–TTA complexes are fully consistent with established Ae β-diketonate chemistry. This analysis reinforces the reliability of the structural assignments and highlights the systematic evolution of coordination geometry across the Ae series, which is central to the structure–property relationships discussed in this work.

In our attempts to obtain the single-crystal structure of the Mg–TTA complex, we strictly followed the same synthetic procedure and crystallization conditions that were successful for the Ca, Sr, and Ba analogues. Despite these efforts, the Mg^2+^ complex consistently yielded only microcrystals of needlelike habit, as shown in Figure S2. These needle crystals were too small and fragile to provide reliable single-crystal diffraction data, which prevented us from resolving the full structure. However, partial indexing of the diffraction pattern was possible, corresponding to approximately 60% of the reflections. The material was assigned to the triclinic crystal system with the following cell parameters: *a* = 9.980 Å, *b* = 12.053 Å, *c* = 17.549 Å, α = 100.95°, β = 98.17°, and γ = 101.81°, and V = 1992.34(4) Å^3^.

The crystalline structure of Ca also exhibits soft noncovalent interactions S···F with distances of 3.109(4) Å, which are consistent with other literature reports that have analyzed the effect on the values of stability constants and the bulk properties of some materials (Figure S1).^66,67^ To summarize, the structural differences among the three complexes can be explained by metal size: the Ba derivative has a slightly expanded lattice and longer M–O distances, while the Sr analog is more compact, and for the Ca complex, the same trend is observed with even shorter Ca–O bonds (Figure 1 and Table 1).

### 3.2 Powder X-ray Diffraction

Powder X-ray Diffraction (PXRD) shown in Figure 3 were analyzed and conducted both experimentally and calculated from single-crystal data to determine main structural parameters (crystallite size) and to validate pertinent structural features such as crystallite size (Scherrer equation),^68^ microstrain (Williamson–Hall method),^69^ and dislocation density (Williamson–Smallman approach)^70^ as summarized in Table 3. The most intense and well-resolved peak (indicated by * in Figure 3) was used to estimate these structural parameters. PXRD has proven to be an informative technique for characterizing the long-range order and structural coherence of these coordination compounds. The peaks present in the low-angle region, which are typical of layered or lamellar organization, suggest that supramolecular interactions between 2-MeImH^+^ and carbonyl or enol moieties from TTA are retained, as described by SCXRD.

**Figure 3:**
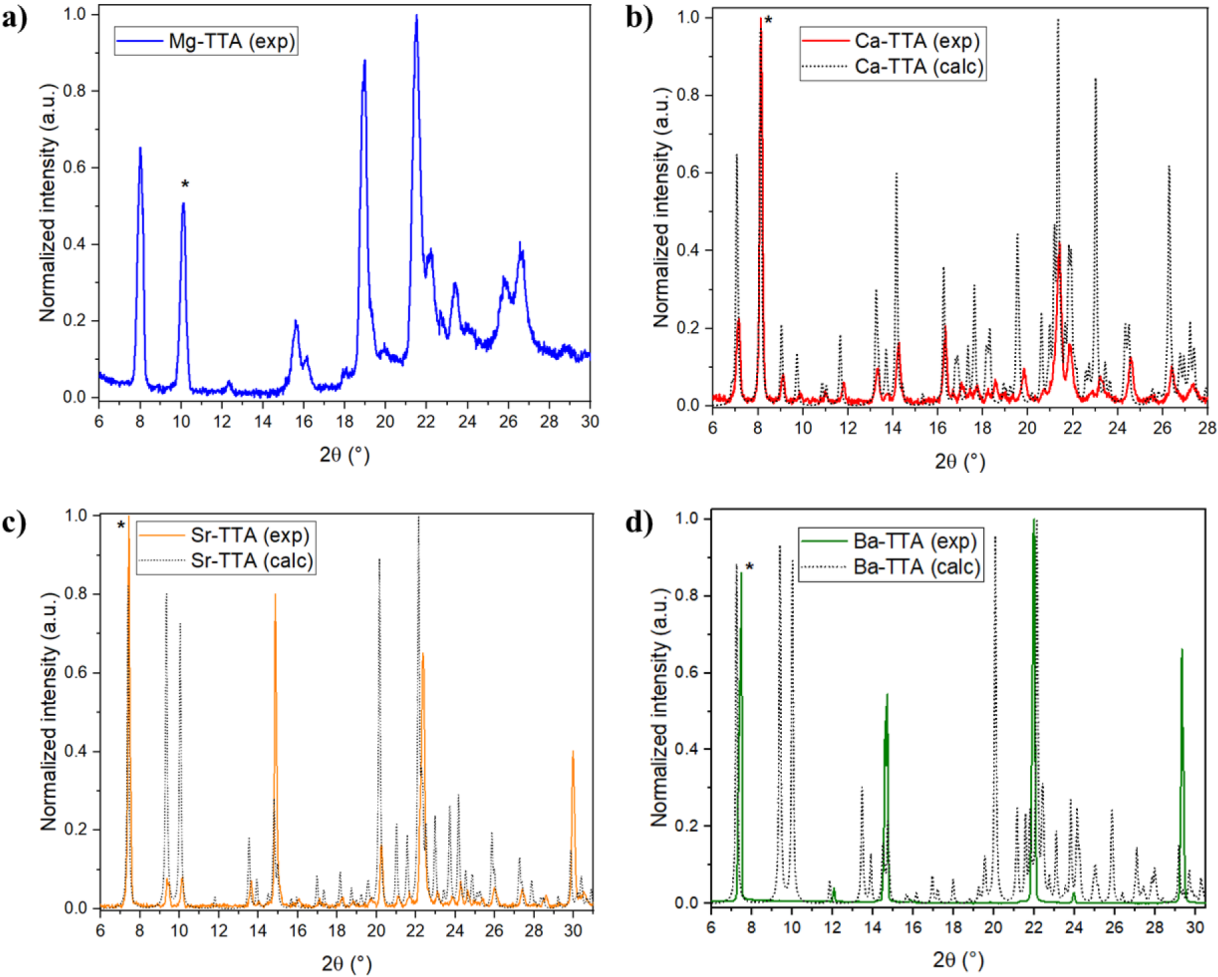
PXRD patterns of the Ae–TTA complexes: **a)** Mg–TTA, **b)** Ca–TTA, **c)** Sr–TTA, and **d)** Ba–TTA.

**Table 3:** Structural Parameters: Average Crystallite Size from the Scherrer Equation (L), Dislocation Density (*δ*), and Micro-Strain (*ε*) of Pure Synthesized Ae–TTA Complexes

**Structural parameter**	**Mg–TTA**	**Ca–TTA**	**Sr–TTA**	**Ba–TTA**
**L (nm)**	26.857	44.646	63.472	136.325
**δ** **(nm** ^**–2**^ **× 10** ^**–3**^ **)**	1.350	0.467	0.225	0.077
**ε (× 10** ^**–3**^ *)*	17.111	9.178	6.405	3.513

PXRD pattern of Mg–TTA shows relatively broad and weak reflections (Figure 3a), indicative of poor crystallinity and very small coherent domain size, and Scherrer analysis does indeed reveal a low average crystallite size (L) of 26 nm (Table 3), which is significantly smaller than in the heavier analogues. The broad, low-intensity peaks suggest that Mg–TTA forms only limited lattice repeats before coherence is lost. The relatively large peak width is also attributed to the large microstrain (ε) of the Mg–TTA lattice, indicating a high level of lattice distortion or disorder in these tiny crystallites. As is usually the case, the dislocation density (δ) is found to be the highest in Mg–TTA, as a small crystallite size is generally coupled with a high density of dislocations and defects.

Experimental PXRD patterns of the bulk materials were compared with patterns calculated from SCXRD structural data, finding that Ca–TTA, Sr–TTA, and Ba–TTA show peaks that are sharper and narrower than for Mg–TTA, reflecting better crystallinity and a larger coherence length relative to Mg–TTA. The overall trend is that the PXRD analyses of Mg–, Ca–, Sr–, and Ba–TTA show that the crystallite size and lattice order increase with increasing ionic radius of the central metal, whereas microstrain and dislocation density decrease, which implies that the heavier Ae cations are more efficient at forming extended, ordered crystal lattices. Whereas the smallest cation (Mg^2+^) yields highly microcrystalline and disordered solids.

The inability of Mg^2+^ to form extensive coordination networks (because of its small radius and high charge density) limits crystal growth, and only needlelike microcrystals with high internal strain are produced; in contrast, Ca^2+^, Sr^2+^, and especially Ba^2+^ (with their larger ionic sizes and higher coordination numbers) support stronger intermolecular interactions and three-dimensional lattice formation (Figure S1), with much larger coherent domains and improved crystallinity; these structure–property correlations are consistent with single-crystal X-ray diffraction observations in which only the Ca, Sr, and Ba complexes yielded well-resolved crystal structures, whereas the Mg complex did not, indicating that increased lattice coherence (and reduced strain/defect levels) accompanies the complexes of larger ionic radius.

Comparison of experimental PXRD with that calculated from the single-crystal structure showed agreement in peak positions but discrepancies in intensities due to preferred orientation and partial amorphization. Despite these intensity mismatches, the single-crystal topology was confirmed to be retained in the bulk material mostly for Ca– and Sr–TTA complexes. However, the extra reflections that appear in the calculated Ba–TTA powder pattern that do not stand out clearly in the experimental PXRD can be explained by the distinction between a perfect structure-factor–based simulation (which shows all symmetry-allowed reflections, many with low intensity) and the real powder measurement. Many of these weak reflections are buried in the background, or overlap with nearby strong peaks, and are therefore not resolved as distinct maxima. The observed intensity discrepancies are attributed to preferred orientation effects arising from anisotropic crystallite morphology, as well as possible microstrain and crystallite size broadening in the powdered sample. Furthermore, small deviations from the ideal single-crystal structure (partial dehydration, minor lattice defects, preferred orientation of plate-like crystals, or a small amorphous fraction) can selectively weaken some reflections and redistribute intensity from those reflections to nearby ones, thus contributing to the extra calculated peaks but not necessarily indicating a phase mismatch.

### 3.3 Scanning Electron Microscopy and Elemental Composition

High-resolution SEM images (Figure 4) reveal characteristic morphologies for each Ae–TTA complex, which depend on the ligand structure, ion type, and growth dynamics. SEM micrographs of Mg–TTA (Figure 4a) show a microcrystalline morphology, with large, irregular agglomerates of particles showing angular, rough surfaces and facets at the edges, indicating the formation of several crystallites that have fused or aggregated in precipitation. Often, these crystal units have high aspect ratios, tending to grow into rod-like or plate-like habits; however, in this case, they form needlelike crystals (Figure S2).

**Figure 4:**
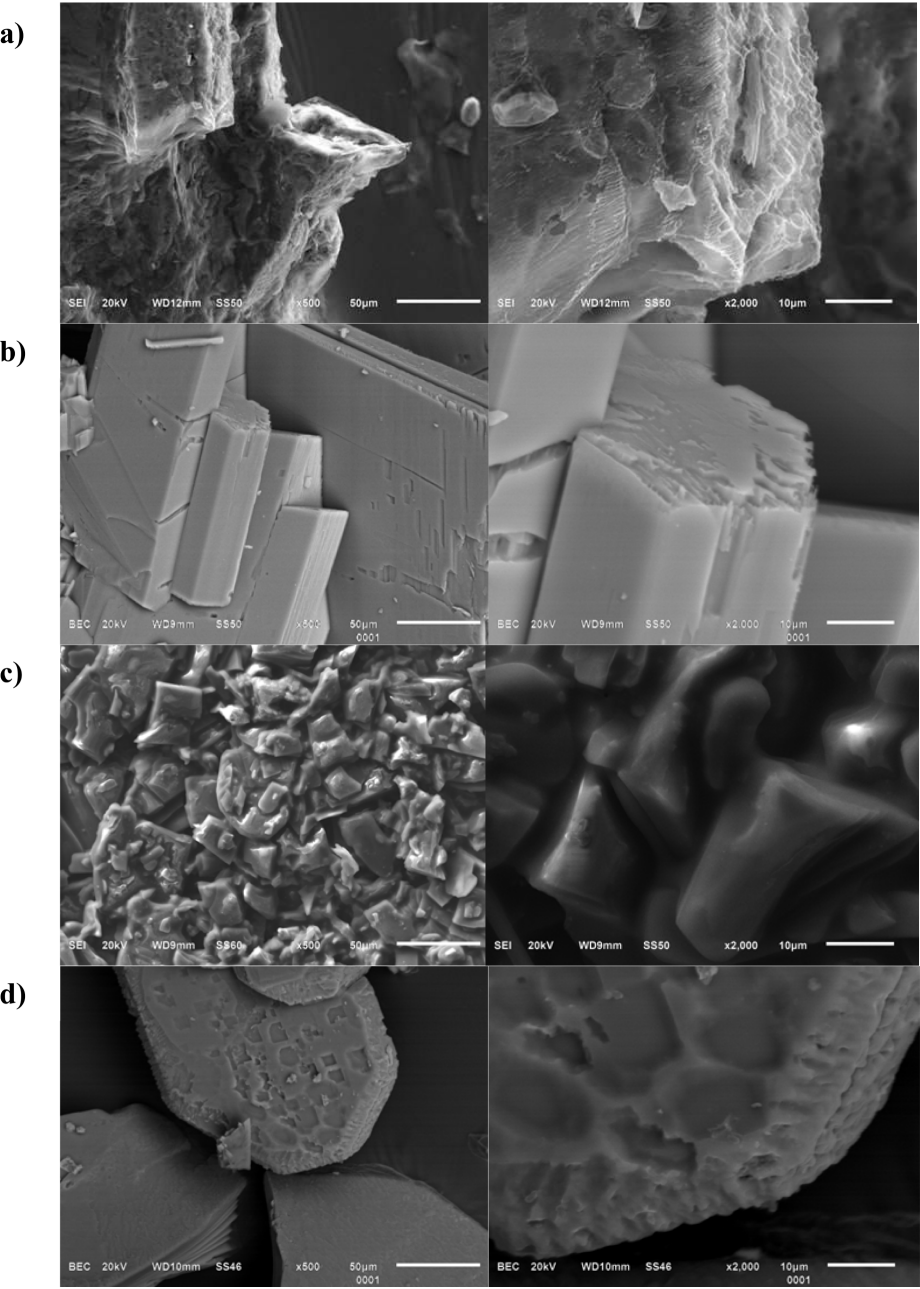
SEM micrographs (×500 and × 2000) and optical image of Ae–TTA complexes: **a)** Mg–TTA, **b)** Ca–TTA, **c)** Sr–TTA, and **d)** Ba–TTA.

The SEM micrographs of the Ca–TTA complex (Figure 4b) show a predominantly block-like morphology that is characterized by large, well-formed, anisotropic crystals composed of well-ordered, platelet-like aggregates with flat facets and sharp edges that are indicative of preferential growth along specific crystallographic directions determined by the coordination environment of the calcium center. The morphological, crystallographic, and structural data are consistent with the notion that Ca–TTA crystallizes as well-ordered, platelet-like aggregates whose growth habit reflects the balance between its seven-coordinate calcium environment and the symmetry constraints of its triclinic lattice.

SEM micrographs (Figure 4c) of the Sr–TTA complex reveal compact aggregates of faceted crystallites, which at low magnification appear as densely packed, intergrown plate and block-like particles with irregular but distinct facets; at higher magnification, individual grains display smooth terraces and blunt edges, consistent with lateral growth and partial coalescence of neighboring crystallites. The absence of coordinated water, which decreases hydrogen-bond-templated layer stacking, allows more efficient packing of the anionic [Sr­(TTA)_4_]^2 –^ units with the imidazolium counterions, resulting in the observed compact, faceted aggregates rather than open, water-textured morphologies observed for the Ca–TTA complex. Crystallographically, the square-prismatic O-donor environment around Sr^2+^ affords a more isotropic coordination envelope than the seven-coordinate Ca^2+^ analogue, which may lower growth anisotropy and promote the formation of stubby plates/blocks that intergrow into compact aggregates.

The SEM micrographs (Figure 4d) of the Ba–TTA complex reveals a morphology that is markedly different from the Ca^2+^ and Sr^2+^ analogues, consistent with the larger Ba^2+^ ionic radius and its coordination environment. The Ba–TTA crystals are large prismatic or block-like particles, often several tens of micrometers in length, and have well-defined faceted edges and a hexagonal or diamond-like cross-section at low magnification; the surfaces of these prisms display relatively smooth faces with localized step-like terraces at higher magnification, indicative of layer-by-layer crystal growth under thermodynamically controlled conditions, compared with the smaller, rougher platelets of Ca–TTA or the aggregated blocks of Sr–TTA, consistent with the PXRD and SCXRD results.

SEM observations could mention that the heavier analogues crystallize as large, well-faceted prisms or plates. In contrast, the lighter analogues are smaller and less regular, as they crystallize as fine, needlelike crystals, forming randomly oriented mats of elongated acicular structures, suggesting a highly anisotropic growth habit; elongation along one crystallographic axis dominates over lateral development. This is consistent with the triclinic symmetry indexed from partial SCXRD data, and the tendency of Mg^2+^, with its smaller ionic radius and high charge density, to adopt coordination environments that limit extended lateral packing.

The morphologies obtained across the series with Ca^2+^ and Mg^2+^ (smaller cations) are more finely divided and aggregated, while Sr^2+^ and Ba^2+^ (larger cations) yield more platy crystals, suggesting that ionic size influences the morphology. While the surfaces are rougher on Sr^2+^ than on Ba^2+^, they are still relatively nonuniform, whereas Ca^2+^ and Mg^2+^ are more irregular. The heavily Ba–TTA shows the most obvious defects, in the form of microsteps and rough edges, and the Sr–TTA crystals are more uniform. Complementing these morphological trends, the EDS analyses confirm the presence of the expected Ae elements in each complex (Figure S3), with metal weight percentages increasing systematically from Mg–TTA (3.95 wt %) to Ca–TTA (6.10 wt %), Sr–TTA (8.99 wt %), and Ba–TTA (11.38 wt %). This monotonic increase reflects the higher atomic masses of the heavier cations and is fully consistent with the stoichiometric composition of the β-diketonate complexes. The more intense metal signals in Sr- and Ba-containing samples also correlate with their larger and more crystalline particles observed in SEM images, which expose broader, compositionally cleaner domains for analysis. The finer and more irregular Mg- and Ca–TTA aggregates yield proportionally lower metal counts due to stronger surface roughness effects and a higher relative contribution of light elements (C, O, F) to the detected signal. Importantly, no extraneous elements were detected beyond trace substrate contributions (e.g., Al from the sample holder), reinforcing the phase purity of the materials across the series. Together, the EDS and SEM results confirm that the morphological evolution observed from Mg^2+^ to Ba^2+^ is accompanied by consistent and predictable changes in elemental ratios, reflecting both the intrinsic metal content and the development of larger, more coherent crystalline domains in the heavier Ae TTA complexes.

### 3.4 Thermal Analysis

Thermogravimetric analysis (TGA) of the Ae–TTA complexes (Figure 5) reveals systematic trends in thermal stability and decomposition behavior across the Ae series. These TGA curves of the Mg–, Ca–, Sr–, and Ba–TTA complexes show decomposition processes associated with progressive removal of coordinated and organic components; however, the thermal behavior is not uniform across the series. Although all complexes ultimately undergo organic ligand degradation followed by formation of metal-rich inorganic residues, their thermal profiles differ significantly in onset temperature, number of discernible steps, and mass-loss distribution. These differences reflect variations in metal–ligand bond strength, coordination geometry, and lattice stabilization across the Ae series.^71^

**Figure 5:**
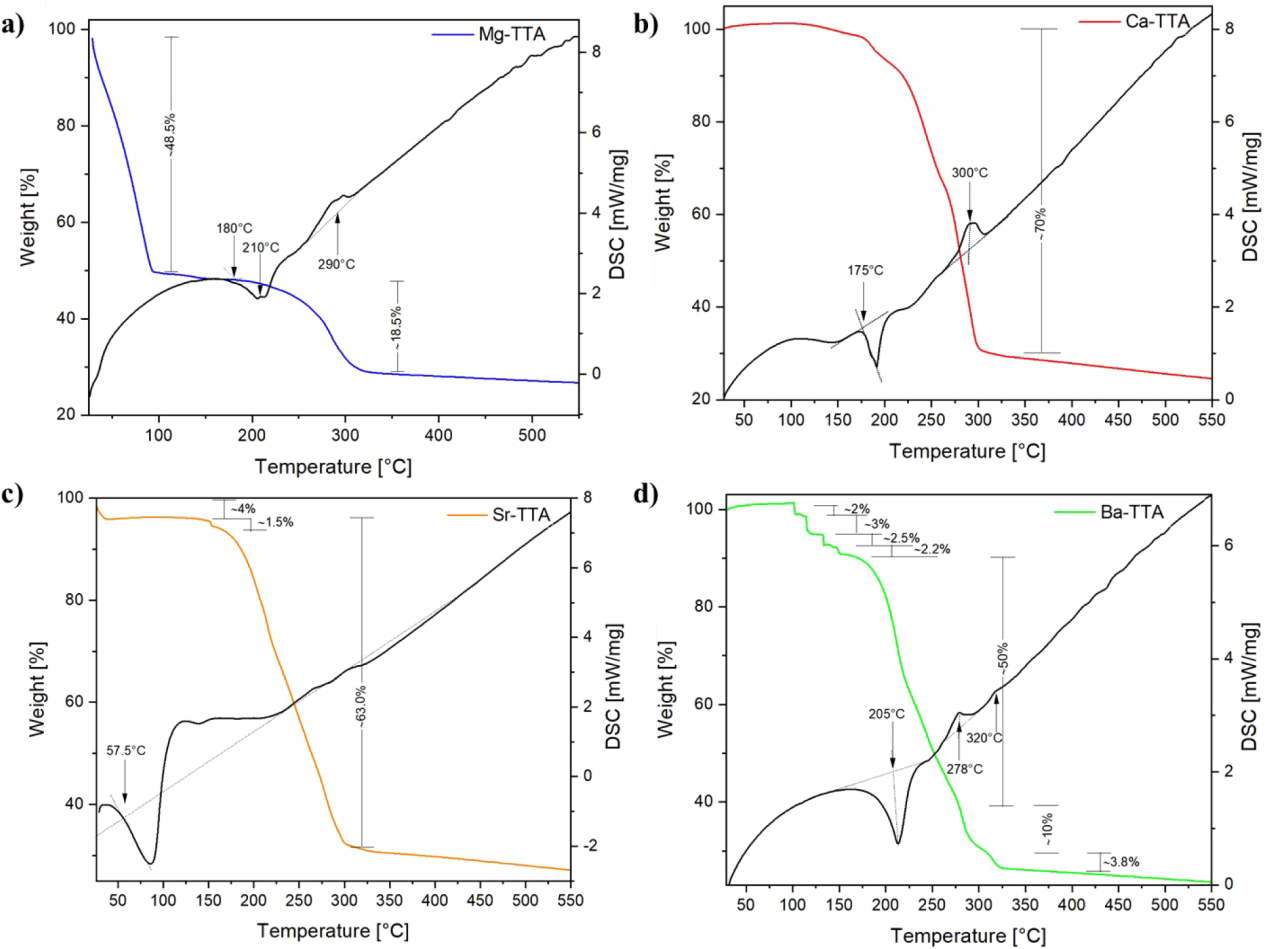
TGA-DSC profiles of: **a)** Mg–TTA, **b)** Ca–TTA, **c)** Sr–TTA, and **d)** Ba–TTA showing thermal stability and decomposition behavior.

For instance, Mg–TTA (Figure 5a) exhibits a pronounced initial mass loss of approximately 48.5% below 110 °C, representing the dominant thermal event. This abrupt decrease suggests removal of weakly bound, labile components or occluded species, such as adsorbed moisture, and partial destabilization of the coordination assembly. A second, more gradual mass loss occurs between 180 and 300 °C, corresponding to decomposition of the β-diketonate framework. Beyond 300 °C, the mass stabilizes, yielding a final residue of 20–23%.

In contrast, Ca–TTA (Figure 5b) shows higher thermal stability at low temperature. Only minor mass loss occurs below 120 °C, which can be attributed to release of coordinated or adsorbed water, in agreement with the crystallographic identification of a bound H_2_O molecule. The main decomposition step occurs between 180 and 260 °C, which accounts for the majority of the organic mass removal. The final residue at 550 °C is around 23–24%.

In case of Sr–TTA, Figure 5c displays a profile very similar to Ca–TTA, but shifted to lower temperature by 10–20 °C. The small initial loss (approximately 4–6%) below 150 °C could correspond to removal of weakly bound species, and the major degradation step between 200 and 300 °C is consistent with breakdown of the TTA ligands. The residual mass (approximately 25–26%) reflects the similar metal content relative to Ca.

Finally, the most clearly resolved multistep behavior is observed for Ba–TTA (Figure 5d), with minor stepwise losses below 180 °C, followed by a dominant decomposition event between 200 and 320 °C. The sharper step resolution suggests sequential destabilization of the coordination environment, consistent with the higher coordination number and expanded lattice of the Ba complex. The final residue (26%) is slightly higher than for the lighter congeners, reflecting the larger atomic mass of Ba.

Overall, Ca– and Sr–TTA exhibit comparable staged degradation pathways, whereas Mg–TTA decomposes at significantly lower temperature and Ba–TTA displays more distinctly separated high-temperature steps. These variations are consistent with differences in metal–oxygen bond distances and lattice organization across the series.

For comparison, the thermal behavior of the free ligands TTA and 2-MeIm is shown in Figure S4. TTA exhibits an endothermic melting transition near 40–50 °C, followed by rapid mass loss commencing above 60 °C and completed by 300 °C, which is consistent with volatilization and decomposition of the molecular β-diketonate. No significant residue remains. 2-MeIm exhibits a melting event near 142–143 °C followed by rapid mass loss completed by 220 °C. Again, negligible residue is observed. The significantly higher residual masses of the Ae–TTA complexes are thus attributed to the inorganic metal component that remains following complete loss of organic ligands.

The modest decrease in thermal stability with increasing metal size is consistent with progressive elongation and weakening of the M-O interactions across the series (Mg > Ca > Sr > Ba).^25,72^ At 550 °C, the remaining masses (23–26%) are consistent with formation of metal-rich inorganic phases based on theoretical mass balance calculations. At these temperatures, complete degradation of organic components is expected, leaving thermodynamically stable Ae oxides and/or carbonate species in the sample holder. Further characterization of the residues was not performed because between 550 and 800 °C there are no changes in TGA nor DSC curves, implying that only oxides and/or carbonates remains stable, consistent with the well-documented decomposition behavior of related Ae β-diketonate systems.^25,71^

The DSC traces support the multistep nature of decomposition. Weak low-temperature endothermic features correspond to minor mass losses observed below 120 °C (release of weakly bound species). The dominant endothermic events between 180 and 300 °C correlate with the TGA mass-loss steps and reflect collective breakdown of the coordination framework and β-diketonate decomposition. The broad temperature ranges indicate overlapping solid-state processes rather than discrete single-bond cleavage events.^71,72^

### 3.5 FTIR Analysis

The IR spectra of the Ae–TTA complexes were analyzed in comparison with the spectra of the free ligands TTA and 2-MeIm (Figures 6 and S5a) to evaluate coordination-induced changes through the characteristic vibrational features of coordinated β-diketonate ligands and the thiophene moiety of TTA together with contributions from the 2-MeImH^+^ counterion and confirm ligand binding modes.

**Figure 6:**
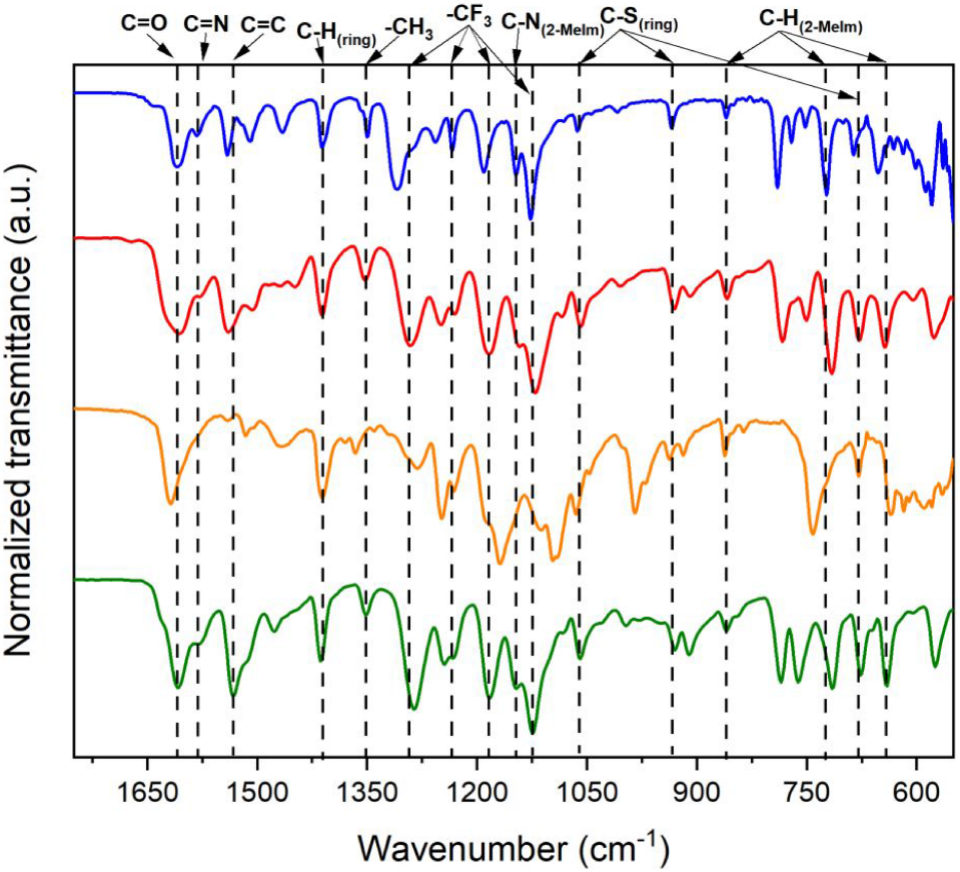
FTIR spectra of the Ae–TTA complexes (**blue**: Mg–TTA, **red**: Ca–TTA, **yellow**: Sr–TTA, and **green**: Ba–TTA).

The IR spectrum of free TTA (Figure S5a) exhibits a strong carbonyl stretching band in the 1650–1680 cm^–1^ region, characteristic of the noncoordinated β-diketone form, together with conjugated ν­(C=C) modes of the enolized system. Intense absorptions in the 1250–1150 cm^–1^ region correspond to ν­(C–F) stretching vibrations of the CF_3_ group. Additional bands near 1060, 930, and 680 cm^–1^ arise from thiophene ν­(C–S) stretching modes, while aromatic C–H bending modes appear between 900 and 750 cm^–1^. The 2-MeIm ligand displays characteristic ν­(C=N) and ν­(C=C) stretching modes in the 1500–1600 cm^–1^ region, along with C–N stretching and ring vibrations between 1200–1000 cm^–1^. These assignments are consistent with literature reports for substituted imidazole systems.^20,73–75^

Upon coordination to the Ae metal centers, significant changes are observed in Ae–TTA spectra (Figure 6) at the β-diketonate region. The strong free-ligand carbonyl band of TTA above 1650 cm^–1^ is absent in the complexes and replaced by a prominent band near 1610 cm^–1^. This band is assigned to the coupled ν­(C=O) and ν­(C=C) stretching modes of the coordinated β-diketonate enolate system. The shift to lower frequency relative to free TTA confirms ligand deprotonation and bidentate chelation to the metal center, in agreement with the crystallographic structures.^73–75^

Bands in the 1580–1530 cm^–1^ range arise from overlapping contributions of ν­(C=N) and ν­(C=C) modes of the imidazolium ring together with conjugated aromatic vibrations of TTA. The persistence of these bands in Ca–, Sr–, and Ba–TTA confirms the presence of the protonated 2-MeImH^+^ counterion observed crystallographically. The CF_3_ group retains its characteristic strong ν­(C–F) absorptions in the 1290–1150 cm^–1^ region across all complexes, indicating that the trifluoromethyl substituent remains chemically intact upon coordination. Minor shifts in this region are attributed to changes in electronic distribution within the coordinated enolate framework rather than alteration of the CF_3_ group itself.^77,78^

The band near 1350 cm^–1^ is assigned primarily to δ­(CH_3_) bending of the 2-MeImH^+^ cation. Additional ring stretching and deformation modes are observed between 1120 and 930 cm^–1^, arising from combined ν­(C–C), ν­(C–N), and aromatic ring vibrations. Thiophene ν­(C–S) stretching modes are retained at 1060, 930, and around 680 cm^–1^ in all complexes, confirming preservation of the heteroaromatic ring during coordination. Aromatic C–H out-of-plane bending vibrations are observed in the 860–750 cm^–1^ region. A relatively intense band observed near 725 cm^–1^ is assigned to imidazolium ring deformation and out-of-plane C–H bending modes, consistent with reported vibrational features of substituted imidazolium systems. The persistence of this band across all complexes provides additional spectroscopic evidence for incorporation of the 2-MeImH^+^ counterion.^76–78^

The high-frequency region (4000–2000 cm^–1^) of the Ae–TTA complexes (Figure S5b) reveals distinct O–H, N–H, and C–H stretching features that differ across the Mg to Ba series. The presence of a broad O–H band exclusively in Ca–TTA is fully consistent with its crystallographically confirmed coordinated water molecule. In contrast, Sr– and Ba–TTA do not contain coordinated water in their crystal structures and accordingly do not display broad hydrogen-bonded O–H absorptions. The sharper bands above 3700 cm^–1^ observed in Mg– and Sr–TTA are characteristic of weakly bound or surface-adsorbed moisture, which is consistent with TGA analysis. Sr–TTA shows a moderately broad feature at 3340 cm^–1^ feature that is attributed to intramolecular N–H···O hydrogen bonding (HB), as discussed in the SCXRD section, which for Ca–TTA and Mg–TTA this band is shifted to 3400 cm^–1^. The weak feature near 2710 cm^–1^ is assigned to combination/overtone (OT) modes. Across all four complexes, the consistent presence of N–H stretching bands in the 3150–3100 cm^–1^ region confirms incorporation of the protonated 2-MeImH^+^ counterion.^74–78^

Across the Mg to Ba series, only minor shifts in band positions are observed, reflecting modest variations in M–O bond lengths and coordination environments rather than changes in ligand binding mode. The overall similarity of the spectra indicates that TTA coordinates in a consistent bidentate fashion in all complexes. The absence of a sharp free TTA carbonyl band above 1650 cm^–1^ and the appearance of the characteristic enolate band near 1610 cm^–1^ provide clear spectroscopic evidence of β-diketonate coordination. Combined with SCXRD data, the IR results confirm successful formation of the Ae–TTA complexes and preservation of the CF_3_ and thiophene functionalities.^75^

IR spectroscopy confirms the presence of β-diketonate ligands coordinated to magnesium, in addition, imidazole-derived species are observed, suggesting the presence of imidazole in the material. On this basis, and considering charge-balance requirements, a tentative formulation involving an anionic magnesium β-diketonate unit stabilized by a protonated imidazolium counterion is proposed. Importantly, this formulation is presented as a compositional hypothesis rather than a structurally resolved model, and no specific coordination number or ligand arrangement around Mg^2+^ is implied.

Such behavior is consistent with the well-documented tendency of magnesium β-diketonate systems to form poorly crystalline or highly aggregated solids, often precluding definitive structural characterization by single-crystal X-ray diffraction. Accordingly, the Mg–TTA compound is discussed here primarily as a comparative reference at the small-ion limit of the Ae series, highlighting the sharp contrast between Mg^2+^ and the heavier Ca^2+^, Sr^2+^, and Ba^2+^ ions, for which well-defined, high-coordination solid-state structures are readily obtained.

The failure to produce large, well-formed single crystals is attributed to the combined effects of the coordination environment and lattice stabilization. Our evidence suggests Mg^2+^ coordinates three bidentate TTA ligands to form an anionic [Mg­(TTA)_3_]^−^ unit charge-balanced by one imidazolium cation as the Ca–TTA complex but not like Mg­(TTA)_2_ complex.^45^ While the Ca–TTA complex is aided by a more tightly packed network of charge-assisted hydrogen bonds, the Mg–TTA lattice has fewer stabilizing interactions, so it is less able to organize a robust three-dimensional packing. Moreover, the small ionic radius and high charge density of Mg^2+^ result in shorter, more polarizing Mg–O bonds, which further increase steric congestion around the bulky TTA ligands and induce packing blocking. All this limits lateral crystal growth and promotes rapid one-dimensional stacking of defect-rich, needlelike microcrystals with high microstrain, leading to Mg–TTA consistently crystallizing as nondiffractable microcrystals, in contrast to the well-developed morphologies of Ca, Sr, and Ba analogues.

## 4 Conclusion

This study demonstrates that the structural, spectroscopic, morphological, and thermal properties of Ae–TTA complexes evolve systematically across the Mg–Ba series as a function of ionic radius. SCXRD establishes that Ca–TTA adopts a seven-coordinate capped trigonal prismatic geometry incorporating a coordinated water molecule, whereas Sr– and Ba–TTA form eight-coordinate square-prismatic environments supported by four bidentate TTA ligands. The progressive increase in average M–O bond distances (Ca–O 2.38 Å to Ba–O 2.74 Å) and expansion of unit-cell volumes reflect the increasing size of the heavier Ae cations. In contrast, Mg–TTA could not be structurally resolved by SCXRD despite repeated crystallization attempts. The material forms microcrystalline needles with limited lattice coherence. Spectroscopic and powder diffraction analyses support β-diketonate coordination and imidazolium incorporation; however, the precise coordination environment around Mg^2+^ remains unresolved and is discussed comparatively rather than structurally.

Infrared spectroscopy confirms consistent enolate coordination of TTA across the series. Only Mg- and Ca–TTA exhibit a broad O–H stretching band consistent with coordinated water, whereas Sr– and Ba–TTA lack such features, in agreement with crystallographic data. All complexes display N–H stretching bands attributable to protonated 2-MeImH^+^ counterions that contribute to lattice stabilization.

Thermal analysis further highlights metal-dependent behavior. Ca– and Sr–TTA show staged ligand decomposition between 180–300 °C, Ba–TTA exhibits more clearly separated high-temperature steps, and Mg–TTA displays significant early mass loss, consistent with a less robust lattice. Residual masses at 550 °C (23–26%) are consistent with formation of thermodynamically stable metal-rich inorganic phases, most plausibly oxides and/or carbonates.

Overall, increasing ionic radius promotes higher coordination numbers, improved lattice coherence, and greater thermal robustness. These findings clarify structure–property relationships in alkaline-earth β-diketonate systems and provide insight into how metal choice governs coordination geometry, crystal growth, and thermal stability in TTA-based complexes.

## 5 Limitations and Future Work

Although this study establishes systematic structural and thermal trends across the Mg–Ba series, certain limitations should be noted. SCXRD data could not be obtained for Mg–TTA, and its coordination environment is therefore inferred from spectroscopic and powder diffraction evidence rather than directly resolved. In addition, while thermogravimetric analysis indicates formation of metal-rich inorganic residues at elevated temperature, definitive phase identification (e.g., oxide versus carbonate composition) was not performed. The present work also focuses primarily on structural and thermal characterization, and photophysical properties were not investigated.

Future studies will address these aspects through detailed phase analysis of thermal residues using high-temperature PXRD or Raman spectroscopy. Steady-state and time-resolved photoluminescence measurements will be conducted to evaluate emission behavior and the influence of coordination geometry and lattice organization. Complementary DFT and TD-DFT calculations will further elucidate electronic structure and excited-state processes, enabling deeper understanding of structure–property relationships in alkaline-earth β-diketonate systems.

## Supplementary Material









## Notes

2476162 Ba–TTA (CIF)
2476165 Sr–TTA (CIF)
2364266­[1] (CIF)
CCDC-2364266, CCDC-2476165, and CCDC-2476162 contain the supplementary crystallographic data for this paper (PDF)

## References

[1] Chapple P. M., Cordier M., Dorcet V., Roisnel T., Carpentier J.-F., Sarazin Y. (2020). A versatile nitrogen ligand for alkaline-earth chemistry. Dalton Trans.

[2] de
Bruin-Dickason C. N., Deacon G. B., Jones C., Junk P. C., Wiecko M. (2019). Functionalised Alkaline Earth Iodides from Grignard
Synthons “PhAeI­(thf)­n” (Ae = Mg-Ba). Eur. J. Inorg. Chem.

[3] Springer. Alkaline-Earth Metal Compounds: Oddities and Applications. Harder, S. ; Ed.; Springer: Berlin, Heidelberg, 2013. Vol. 45. 10.1007/978-3-642-36270-5.

[4] Harder, S. Early main group metal catalysis: Concepts and reactions. Wiley-VCH, 2020.

[5] Moxey G. J., Blake A. J., Lewis W., Kays D. L. (2015). Alkaline Earth Complexes
of a Sterically Demanding Guanidinate Ligand. Eur. J. Inorg. Chem.

[6] Yang D., Ding Y., Wu H., Zheng W. (2011). Synthesis and Structural
Characterization of Alkaline-Earth-Metal Bis­(amido)­silane and Lithium
Oxobis­(aminolato)­silane Complexes. Inorg. Chem.

[7] Raikundliya R., Kalyani N. T., Dhoble S. J. (2025). Exploring the structural, morphological
and luminescence attributes of KMq3: CTAB (M = Mg, Sr, Ca, Ba) phosphor
for optoelectronic applications. J. Opt.

[8] Weller, M. ; Rourke, J. ; Overton, T. ; Armstrong, F. The Group 2 elements. In Inorganic Chemistry. Oxford University Press, 2018. 10.1093/hesc/9780198768128.003.0014.

[9] Zhou M., Frenking G. (2021). Transition-Metal Chemistry
of the Heavier Alkaline
Earth Atoms Ca, Sr, and Ba. Acc. Chem. Res.

[10] Weng Y., Jian Y., Huang W., Xie Z., Zhou Y., Pei X. (2023). Alkaline earth metals for osteogenic scaffolds: From mechanisms to
applications. J. Biomed. Mater. Res., Part B.

[11] Genter, M. B. Magnesium, Calcium, Strontium, Barium, and Radium. In Patty’s Toxicology. Wiley, 2012; pp. 145–166. 10.1002/0471435139.tox028.pub2.

[12] Zhang J. (2023). Magnesium research and
applications: Past, present and future. J. Magnesium
Alloys.

[13] Aikin M. (2025). Recent Advances in Biodegradable Magnesium Alloys for
Medical Implants:
Evolution, Innovations, and Clinical Translation. Crystals.

[14] Khajuria, G. ; Gupta, V. Magnesium Alloys for Biomedical Applications. CRC Press: Boca Raton, 2024, pp. 1–19. 10.1201/9781003400462-1.

[15] Huang Y. (2023). Development and prospects
of degradable magnesium alloys for structural
and functional applications in the fields of environment and energy. J. Magnesium Alloys.

[16] Rafiei S., Habibolahzadeh A., Wiese B. (2024). Environment-COnscious magnesium (ECO-Mg):
A review. Clean. Mater.

[17] Vikulova E. S., Rikhter E. A., Piryazev D. A., Zherikova K. V., Morozova N. B. (2020). Structure of mixed-ligand magnesium dipivaloylmethanate
complexes with propylenediamine and its derivative. J. Struct. Chem.

[18] Otway, D. J. ; Rees, W. S., Jr. Group 2 Element β-Diketonate Complexes: Synthetic and Structural Investigations Coord. Chem. Rev. 2001 32 8 10.1002/chin.200108252

[19] Fenton D. E. (1971). Co-ordinative
saturation of magnesium β-diketonates. J. Chem. Soc. A.

[20] Arunasalam V.-C., Drake S. R., Hursthouse M. B., Malik K. M. A., Miller S. A. S., Mingos D. M. P. (1996). Synthesis, structure
and characterisation of magnesium
and calcium β-diketonate complexes [Ca _3_ (tmhd) _6_] and [Ca _2_ (tmhd) _4_ (EtOH) _2_]­(Htmhd = 2,2,6,6-tetramethylheptane-3,5-dione). J. Chem. Soc. Dalton Trans.

[21] George S. M. (2015). Heteroleptic strontium complexes stabilized by donor-functionalized
alkoxide and β-diketonate ligands. Dalton
Trans.

[22] George S. M., Park B. K., Kim C. G., Chung T. (2014). Heteroleptic Group
2 Metal Precursors for Metal Oxide Thin Films. Eur. J. Inorg. Chem.

[23] Drozdov A., Troyanov S. (1996). The structural chemistry
of iia group metal diketonates. Main Group Met.
Chem..

[24] Maverick A. W. (2023). Structural
Flexibility of Metal Chelate Complexes and Its Relation to Supramolecular
Chemistry. Helv. Chim. Acta.

[25] Guillon H., Hubert-Pfalzgraf L. G., Vaissermann J. (1997). Synthesis, characterization and thermal
behaviour of calcium and strontium 2, 2, 6, 6-tetramethyl-3,5-heptanedionate
complexes. crystal structure of [sr­(μ, η2-thd)­(η2-thd)­(η2-meoc2h4oh)]­2. Main Group Met. Chem..

[26] Thompson S. C., Cole-Hamilton D. J., Gilliland D. D., Hitchman M. L., Barnes J. C. (1992). Stable
and volatile β-diketonate complexes of copper, calcium, strontium,
barium and yttrium for use as chemical vapour deposition precursors. Adv. Mater. Opt. Electron.

[27] Giricheva N. I., Girichev G. V., Belova N. V., Isakova N. A., Kuzmina N. P. (1999). Structure
and energetics of β-diketonates. IX. Molecular structure of
Sr­(DPM)­2 according to gas phase electron diffraction data. J. Struct. Chem.

[28] Panda T. K., Zulys A., Gamer M. T., Roesky P. W. (2005). Bis­(phosphinimino)­methanides
as ligands in divalent lanthanide and alkaline earth chemistry –
synthesis, structure, and catalysis. J. Organomet.
Chem.

[29] Gärtner M., Görls H., Westerhausen M. (2007). Synthesis and Molecular Structures
of Phenylamides of Magnesium, Calcium, Strontium, and Barium –
From Molecular to Polymeric Structures. Inorg.
Chem.

[30] Bacsa J., Ramírez-Palma L. G., Cortés-Guzmán F., Wallen C. M., Scarborough C. C. (2018). An Examination of the Electron Densities
in a Series of Tripodal Cobalt Complexes Bridged by Magnesium, Calcium,
Strontium, and Barium †. Crystals.

[31] Liu X. (2024). Exploring the Use of
‘Honorary Transition Metals’ To
Push the Boundaries of Planar Hypercoordinate Alkaline-Earth Metals. J. Am. Chem. Soc.

[32] Blois L. (2024). Unusually Large Ligand
Field Splitting in Anionic Europium­(III) Complexes
Induced by a Small Imidazolic Counterion. Inorg.
Chem.

[33] Culeac I. P. (2022). Synthesis and Characterization of Coordination
Compound [Eu­(μ2-OC2H5)­(btfa)­(NO3)­(phen)]­2phen
with High Luminescence Efficiency. Nanomaterials.

[34] Medina-Velazquez D.-Y., Osorio-de-la-Rosa E., Colín Calderón V. H., García Murillo A., Carrillo Romo F.-d.-J., Ruiz Guerrero M. d. R. (2022). Evaluation of the morphology and
chelating agent excess in the design of luminescent metalorganic framework
of europium thenoyltrifluoroacetone. Adv. Mech.
Eng.

[35] Rajhans V., Kalyani N. T., Ugale A., Dhoble S. J. (2024). Exploring the Feasibility
of Eu­(TTA)­3TPPO as Pigment for Fluorescent Paint. J. Opt. Photonics Res.

[36] Tang Q. (2022). Novel Cuboid-like Crystalline Complexes (CLCCs), Photon
Emission,
Fluorescent Fibers, and Bright Red Fabrics of Eu3+ Complexes Adjusted
by Amphiphilic Molecules. Polymers.

[37] Bordian, O. ; Verlan, V. ; Iovu, M. ; Culeac, I. ; Zubareva, V. ; Enachescu, M. ; Bojin, D. ; Siminel, A. Photoluminescence Properties of Eu­(TTA)­3­(Ph3PO)­2, In 5th International Conference on Nanotechnologies and Biomedical Engineering Conference paper Springer 2022; pp 84–91. 10.1007/978-3-030-92328-0_12.

[38] Khan L. U., Khan Z. U., Blois L., Tabassam L., Brito H. F., Figueroa S. J. A. (2023). Strategy to Probe
the Local Atomic Structure of Luminescent
Rare Earth Complexes by X-ray Absorption Near-Edge Spectroscopy Simulation
Using a Machine Learning-Based PyFitIt Approach. Inorg. Chem.

[39] Costa I. F. (2024). Luminescence properties of lanthanide tetrakis
complexes as molecular
light emitters. Coord. Chem. Rev.

[40] Miyazaki S., Gotanda M., Kitagawa Y., Hasegawa Y., Miyata K., Onda K. (2024). Full Picture of Energy
Transfer in a Trivalent Europium Complex with
Bidentate β-Diketonate Ligand. J. Phys.
Chem. Lett.

[41] Silva R. A. N. (2024). [Eu­(tta)­3­(Phen-derived)] complexes: Theoretical
and
empirical approaches enlightening their photophysical behavior. J. Lumin.

[42] Bravo-Arredondo J. M., Bernès S., Mejía K., Anzaldo B., Ramírez E., Moro D. (2025). 2-Methylimidazolium tetrakis­(2-thenoyltrifluoroacetonato-κ ^2^
*O*, *O* ′)­neodymium­(III). IUCrData.

[43] Xu H., Tan Y., Hou Z., Fu C., Lin L.-R. (2022). Insights
into the
Effect of Trans-to-Cis Photoisomerization of a Co-coordinated Stilbene
Derivative on the Luminescence of Di-β-diketonate Lanthanide
Complexes. ACS Omega.

[44] Xu, H. ; Chen, R. ; Sun, Q. ; Lai, W. ; Su, Q. ; Huang, W. ; Liu, X. Recent Progress in Metal—Organic Complexes for Optoelectronic Applications Chem. Soc. Rev. 2014 45 26 10.1002/chin.201426289 24531130

[45] Kuratieva N. V., Vikulova E. S., Zherikova K. V. (2018). Crystal
Chemistry Study of Two Magnesium
Complexes with Trifluoroacetylacetone. J. Struct.
Chem.

[46] Park C., Choi H., Lee G. Y., Park B. K., Ryu J. Y., Chung T.-M. (2023). Synthesis of Mononuclear Strontium
Complexes with Polyether
and β-Diketonato Ligands. ACS Omega.

[47] Kuzmina N. (2009). Novel Low Melting Point Barium and Strontium Precursors for the MOCVD
Growth of Barium-Strontium-Titanate Films. Chem.
Vap. Deposition.

[48] Gordon R. G., Barry S. T., Liu X., Teff D. J. (1999). Liquid Compounds
for CVD of Alkaline Earth Metals. MRS Proc.

[49] Hanusa, T. P. ; Bierschenk, E. J. ; Engerer, L. K. ; Martin, K. A. ; Rightmire, N. R. Alkaline Earth Chemistry: Synthesis and Structures. In Comprehensive Inorganic Chemistry II. Elsevier, 2013; pp. 1133–1187. 10.1016/B978-0-08-097774-4.00145-5.

[50] Vikulova E. S., Piryazev D. A., Zherikova K. V., Alferova N. I., Morozova N. B., Igumenov I. K. (2013). Crystal structure
of two complexes containing tris-(β-diketonato)­magnate
anion. J. Struct. Chem.

[51] Drake S. R., Hursthouse M. B., Malik K. M. A., Miller S. A. S. (1993). The synthesis
and X-ray structure characterisation of the volatile complexes [Sr­(thd)­2­{Me­(OCH2CH2)­3OMe}]
and [Sr2­(thd)­4­{Me­(OCH2CH2)­2OMe}­2­(μ-H2O)]­(Hthd = 1,1,1,6,6,6-hexamethylheptane-2,4-dione). J. Chem. Soc., Chem. Commun.

[52] Park C. (2021). Synthesis and Crystal
Structures of New Strontium Complexes with
Aminoalkoxy and β-Diketonato Ligands. ACS Omega.

[53] Jiménez G. L., Rosales-Hoz M. J., Leyva M. A., Reyes-Rodríguez J. L., Galindo-García U., Falcony C. (2021). Structural analysis
of an Europium-Sodium complex containing 2-thenoyltrifluoroacetone
and succinimide as ligands, a highly photoluminescent material. J. Mol. Struct.

[54] Cao R., Nishiyama R., Nakamura K., Kobayashi N. (2025). Luminescent
Hybrid Material Based on the Europium­(III)−β-Diketone
Complex Doped with Smectite. Macromol. Chem.
Phys.

[55] Lu P. (2020). Tb3+/Eu3+
Complex-Doped Rigid Nanoparticles in Transparent Nanofibrous
Membranes Exhibit High Quantum Yield Fluorescence. Nanomaterials.

[56] Sheldrick G. M. (2015). Crystal
structure refinement with SHELXL. Acta Crystallogr.,
Sect. C: struct. Chem.

[57] Putz, H. ; Brandenburg, K. Diamond - Crystal and Molecular Structure Visualization, Crystal Impact Accessed 16 January 2025.

[58] Krieck S., Görls H., Westerhausen M. (2010). Mechanistic
Elucidation of the Formation
of the Inverse Ca­(I) Sandwich Complex [(thf) _3_ Ca­(μ-C _6_ H _3_ −1,3,5-Ph _3_)­Ca­(thf) _3_] and Stability of Aryl-Substituted Phenylcalcium Complexes. J. Am. Chem. Soc.

[59] Paolini T. B. (2023). The influence of imidazolium
counterions on the luminescence properties
of Cnmim­[Eu­(tta)­4] tetrakis complexes in solid-state and ionic liquid
solutions. J. Lumin.

[60] Sahbari J. J., Olmstead M. M. (1983). Structure of cis-bis­(acetylacetonato)­diaquacalcium
monohydrate, [Ca­(C5H7O2)­2­(H2O)­2].H2O. Acta Crystallogr.,
Sect. C: Cryst. Struct. Commun..

[61] Darr J. A., Drake S. R., Hursthouse M. B., Abdul Malik K. M., Miller S. A. S., Mingos D. M. P. (1997). Monomeric Group
2 metal β-diketonates
stabilised by protonated amine ligands as tight cation–anion
pairs; crystal structure of [Htmen]­2­[Sr­(tfpd)­4] (tfpd = 1,1,1-trifluoropentane-2,4-dionate,
tmen = N,N,N′,N′ -tetramethylethane-1,2-diamine). J. Chem. Soc. Dalton Trans.

[62] Drake S. R., Miller S. A. S., Hursthouse M. B., Malik K. M. A. (1993). Monomeric strontium
and barium β-diketonate adducts with polyethers; the X-ray crystal
structures of [Sr­(Ph2ACAC)­2­(tetraglyme)] and [Ba­(THD)­2­(tetraglyme)]. Polyhedron.

[63] Hollander F. J., Templeton D. H., Zalkin A. (1973). Investigations of alkaline-earth
β-diketone complexes. III. The crystal and molecular structure
of bis­(1,3-diphenyl-1,3-propanedionato)strontium hemiacetonate. Acta Crystallogr., Sect. B: Struct. Crystallogr. Cryst. Chem.

[64] Drozdov A., Troyanov S. (1993). Synthesis and X-ray crystal structures of tetrakis-(2,2,6,6-tetramethylheptane-3,5-dionato)­bis­(2,2′-bipyridyl)­dibarium
Ba2­(thd)­4­(bipy)­2 and catena-[dodecakis­(2,2-dimethylpropanoato)­bis­(2,2,6,6-tetramethylheptane-3,5-dionato)­tetra­(pyridine)­tetra­(aqua)
heptabarium] pyridine solvate {Ba7­(piv)­12­(thd)­2­(py)­4­(H2O)­4·2py}. Polyhedron.

[65] Rees W. S., Carris M. W., Hesse W. (1991). Synthesis
and X-ray diffraction crystal
structure of bis­[bis­(2,2,6,6-tetramethylheptane-3,5-dionato)­diamminebarium].
A novel low-molecularity barium compound. Inorg.
Chem.

[66] Bauer M. R. (2016). Harnessing Fluorine–Sulfur Contacts and Multipolar Interactions
for the Design of p53 Mutant Y220C Rescue Drugs. ACS Chem. Biol.

[67] Thorley K. J., McCulloch I. (2018). Why are S–F
and S–O non-covalent interactions
stabilising?. J. Mater. Chem. C.

[68] Mustapha S. (2019). Comparative study of
crystallite size using Williamson-Hall and Debye-Scherrer
plots for ZnO nanoparticles. Adv. Nat. Sci.:
Nanosci. Nanotechnol.

[69] Mote V., Purushotham Y., Dole B. (2012). Williamson-Hall analysis
in estimation
of lattice strain in nanometer-sized ZnO particles. J. Theor. Appl. Phys.

[70] Williamson G. K., Smallman R. E. (1956). III. Dislocation
densities in some annealed and cold-worked
metals from measurements on the X-ray Debye-Scherrer spectrum. Philos. Mag.

[71] Moitinho A. B. S., Ionashiro E. Y., de Souza G. R., Fertonani F. L. (2004). Solid-state
chelates of ethylenediamine- tetraacetate with alkali earth metals. J. Therm. Anal. Calorim.

[72] Zaafarany I., Khairou K., Tirkistani F., Iqbal S., Khairy M., Hassan R. (2012). Kinetics and Mechanism
of Non-Isothermal Decomposition
of Ca­(II)-, Sr­(II)- and Ba (II)- Cross-Linked Divalent Metal-Alginate
Complexes. Int. J. Chem.

[73] Vasilyeva A. A., Glazunova T. Y., Tereshchenko D. S., Lermontova E. K. (2021). A novel
calcium trifluoroacetate structure. Fine Chem.
Technol.

[74] Li B. (2016). Insight into the roles
of structures and energy levels of mono- and
bis-β-diketones on sensitizing Nd­(iii) NIR-luminescence. Dalton Trans.

[75] Mirtamizdoust B. (2024). Exploring C–F···π
Interactions: Synthesis,
Characterization, and Surface Analysis of Copper β-Diketone
Complexes. ACS Omega.

[76] Wang G.-F. (2014). Synthesis and structural
characterization of two copper­(II) complexes
constructed from copper­(II) thenoyltrifluoroacetonate and the rigid
imidazolyl-based ligands. Crystallogr. Rep..

[77] Foreman M. M., Terry L. M., Weber J. M. (2023). Binding Pocket Response of EDTA Complexes
with Alkaline Earth Dications to Stepwise Hydration—Structural
Insight from Infrared Spectra. J. Phys. Chem.
A.

[78] Yamada T., Mizuno M. (2018). Characteristic Spectroscopic Features
because of Cation–Anion
Interactions Observed in the 700–950 cm–1 Range of Infrared
Spectroscopy for Various Imidazolium-Based Ionic Liquids. ACS Omega.

