# Supplementary material for: Coordination Geometry and Structure–Property Relationships in Alkaline-Earth β‑Diketonate Complexes with 2‑Methylimidazole
Source: ACS Omega. 2026 May 6;11(19):28703–16. doi: 10.1021/acsomega.5c12982 (PMC13191693; doi:10.1021/acsomega.5c12982)
Supplement: Supplementary file 4 [file ao5c12982_si_004.pdf]

SUPPLEMENTARY INFORMATION

Coordination Geometry and Structure-Property Relationships in Alkaline Earth β-Diketonate Complexes with 2-Methylimidazole

José Manuel Bravo-Arredondo[1]*, Anayeli Carrasco-Ruiz[2], José Andrés Reyes-Avendaño[3], María Josefina Robles-Águila[1], Dulce Yolotzin Medina-Velázquez[2], Efrain Rubio Rosas[4].

[1] Benemérita Universidad Autónoma de Puebla, Instituto de Ciencias, Centro de Investigación en Dispositivos Semiconductores, Edificio 105 C, Boulevard 14 Sur y Av. San Claudio, Col. San Manuel, C. P. 72570, Puebla, Pue., México.

[2] Universidad Autónoma de Tlaxcala, Facultad de Ciencias Básicas, Ingeniería y Tecnología, De Apizaquito, 20 de Noviembre, 90401, Apizaco, Tlaxcala, México.

[3] Benemérita Universidad Autónoma de Puebla, Facultad de Ingeniería Química, Ciudad Universitaria, Av. San Claudio y 18 Sur, Col. Jardines de San Manuel, C. P. 72570, Puebla, Pue., México.

[4] Benemérita Universidad Autónoma de Puebla, Centro Universitario de Vinculación y Transferencia de Tecnología, Benemérita Universidad Autónoma de Puebla, CP 72570, Puebla, México.

*Corresponding Author Email: col471344@colaborador.buap.mx

Table S1: Selected bond lengths for Ca-, Sr-, and Ba-TTA complexes.

Ca-TTA		Sr-TTA		Ba-TTA	
**Bond**	**Bond Length**	**Bond**	**Bond Length**	**Bond**	**Bond Length**
Ca(1)-O(2)	2.328(3)	Sr(1)-O(1)	2.569(3)	Ba(1)-O(1)	2.709(3)
Ca(1)-O(3)	2.377(3)	Sr(1)-O(3)	2.583(3)	Ba(1)-O(3)	2.728(3)
Ca(1)-O(4)	2.379(4)	Sr(1)-O(4)	2.590(3)	Ba(1)-O(5)	2.739(2)
Ca(1)-O(1)	2.384(3)	Sr(1)-O(2)	2.645(3)	Ba(1)-O(4)	2.793(3)
Ca(1)-O(6)	2.393(3)	S(1)-C(19)	1.667(7)	S(1)-C(4)	1.684(5)
Ca(1)-O(5)	2.396(3)	S(1)-C(16)	1.682(5)	S(1)-C(1)	1.697(4)
Ca(1)-O(7)	2.457(3)	S(2)-C(6)	1.690(6)	S(2)-C(12)	1.683(6)
S(1)-C(2)	1.654(6)	S(2)-C(5)	1.696(5)	S(2)-C(9)	1.688(5)
S(1)-C(6)	1.681(5)	F(1)-C(4)	1.314(6)	F(1)-C(8)	1.321(7)
S(2)-C(5)	1.665(7)	F(2)-C(4)	1.316(6)	F(2)-C(8)	1.310(6)
S(2)-C(9)	1.688(5)	F(3)-C(4)	1.307(7)	F(3)-C(8)	1.312(6)
S(3)-C(18)	1.696(6)	F(4)-C(12)	1.362(7)	F(4)-C(16)	1.284(5)
S(3)-C(15)	1.699(4)	F(5)-C(12)	1.286(6)	F(5)-C(16)	1.285(6)
F(1)-C(22)	1.311(7)	F(6)-C(12)	1.290(6)	F(6)-C(16)	1.370(7)
F(2)-C(22)	1.268(6)	O(1)-C(1)	1.256(5)	O(1)-C(13)	1.234(5)
F(3)-C(20)	1.331(6)	O(2)-C(3)	1.274(5)	O(3)-C(5)	1.251(4)
F(4)-C(13)	1.250(7)	O(3)-C(9)	1.241(5)	O(4)-C(7)	1.265(5)
F(5)-C(22)	1.320(7)	O(4)-C(11)	1.269(5)	O(5)-C(15)	1.266(4)
F(7)-C(20)	1.338(7)	N(1)-C(21)	1.328(7)	N(1)-C(17)	1.329(6)
F(8)-C(20)	1.291(7)	N(1)-C(22)	1.346(7)	N(1)-C(18)	1.362(7)
F(9)-C(13)	1.337(12)	N(1)-H(2)	0.89(6)	N(2)-C(17)	1.324(6)
F(10)-C(13)	1.222(8)	N(2)-C(21)	1.324(7)	N(2)-C(19)	1.363(7)
O(1)-C(1)	1.255(5)	N(2)-C(23)	1.356(7)	C(1)-C(2)	1.406(5)
O(2)-C(8)	1.240(5)	N(2)-H(1A)	0.86(7)	C(1)-C(5)	1.477(5)

O(3)-C(12)	1.252(5)	C(1)-C(2)	1.431(6)	C(2)-C(3)	1.401(6)
O(4)-C(14)	1.248(5)	C(1)-C(5)	1.471(6)	C(3)-C(4)	1.326(7)
O(5)-C(19)	1.274(5)	C(2)-C(3)	1.356(6)	C(5)-C(6)	1.418(5)
O(6)-C(21)	1.264(5)	C(2)-H(1)	0.93	C(6)-C(7)	1.369(5)

Table S2: Selected bond angles for Ca-, Sr-, and Ba-TTA complexes.

**Ca-TTA**		**Sr-TTA**		**Ba-TTA**	
**Angle**	**Bond Angle (°)**	**Angle**	**Bond Angle (°)**	**Angle**	**Bond Angle (°)**
O(2)-Ca(1)-O(#3)	115.51(11)	O(1)-Sr(1)-O(3)	71.69(10)	O(1)-Ba(1)-O(3)	73.79(9)
O(2)-Ca(1)-O(4)	76.20(13)	O(1)-Sr(1)-O(4)	74.74(9)	O(1)-Ba(1)-O(5)	64.90(8)
O(3)-Ca(1)-O(4)	75.40(11)	O(3)-Sr(1)-O(4)	67.99(9)	O(3)-Ba(1)-O(5)	75.86(9)
O(2)-Ca(1)-O(#1)	157.09(12)	O(1)-Sr(1)-O(2)	65.90(9)	O(1)-Ba(1)-O(4)	70.19(9)
O(3)-Ca(1)-O(1)	74.16(11)	O(3)-Sr(1)-O(2)	68.26(10)	O(3)-Ba(1)-O(4)	62.55(8)
O(4)-Ca(1)-O(#1)	126.67(11)	O(4)-Sr(1)-O(2)	74.72(8)	O(5)-Ba(1)-O(4)	75.62(8)
C(2)-S(1)-C(6)	93.2(3)	O(1)-Sr(1)-O(#3)	108.31(10)	O(1)-Ba(1)-O(#3)	106.21(9)
C(5)-S(2)-C(9)	92.7(3)	O(1)-Sr(1)-O(#4)	105.26(9)	O(1)-Ba(1)-O(#5)	115.10(8)
C(18)-S(3)-C(15)	91.5(2)	O(3)-Sr(1)-O(#4)	112.01(9)	O(3)-Ba(1)-O(#5)	104.14(9)
C(25)-N(1)-C(26)	108.3(4)	O(1)-Sr(1)-O(#2)	114.10(9)	O(1)-Ba(1)-O(#4)	109.81(9)
C(25)-N(2)-C(27)	110.3(4)	O(3)-Sr(1)-O(#2)	111.74(10)	O(3)-Ba(1)-O(#4)	117.45(8)
C(25)-N(2)-H(2)	124.80	O(4)-Sr(1)-O(#2)	105.72(8)	O(5)-Ba(1)-O(#4)	104.08(8)
C(27)-N(2)-H(2)	124.80	C(19)-S(1)-C(16)	94.1(3)	C(4)-S(1)-C(1)	91.7(2)
F(10)-C(13)-F(4)	111.6(7)	C(6)-S(2)-C(5)	91.9(3)	C(12)-S(2)-C(9)	93.4(3)
F(10)-C(13)-F(9)	100.1(9)	C(21)-N(1)-C(22)	109.4(4)	C(17)-N(1)-C(18)	109.3(4)
F(4)-C(13)-F(9)	102.1(8)	C(21)-N(1)-H(2)	117(4)	C(17)-N(2)-C(19)	109.2(4)
F(10)-C(13)-C(12)	113.7(6)	C(22)-N(1)-H(2)	133(4)	N(2)-C(17)-N(1)	107.4(5)
		C(21)-N(2)-C(23)	109.4(4)	N(2)-C(17)-C(20)	127.5(5)

		C(21)-N(2)-H(1A)	125(4)	N(1)-C(17)-C(20)	125.1(4)
		F(3)-C(4)-F(1)	106.9(5)	F(2)-C(8)-F(3)	106.7(5)
		F(3)-C(4)-F(2)	105.6(5)	F(2)-C(8)-F(1)	106.8(5)
		F(1)-C(4)-F(2)	106.4(5)	F(3)-C(8)-F(1)	105.4(5)
		F(3)-C(4)-C(3)	110.6(4)	F(2)-C(8)-C(7)	114.9(4)

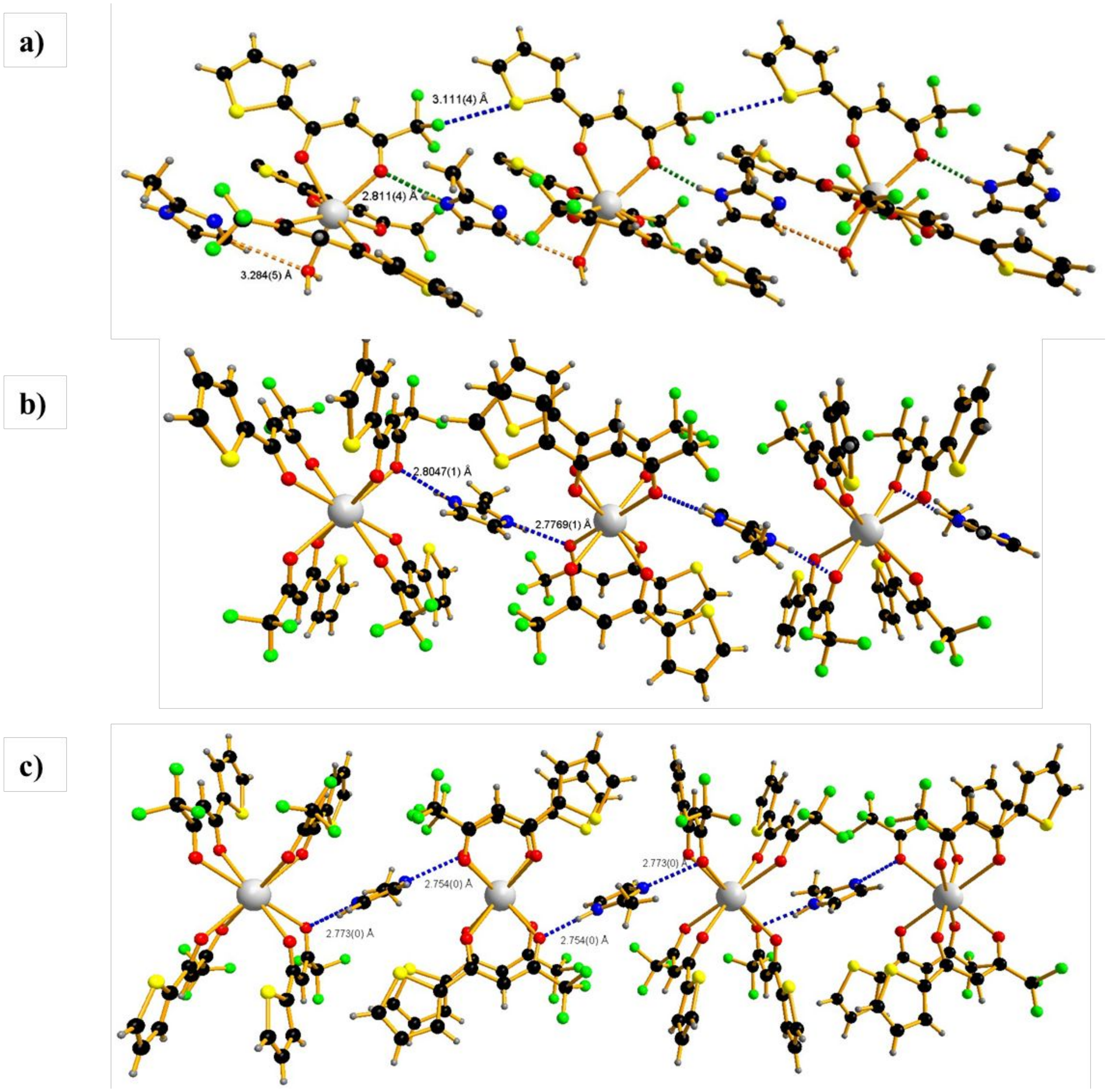

Figure S1. Supramolecular interactions of **a)** Ca-, **b)** Sr-, and **c)** Ba-TTA complexes.

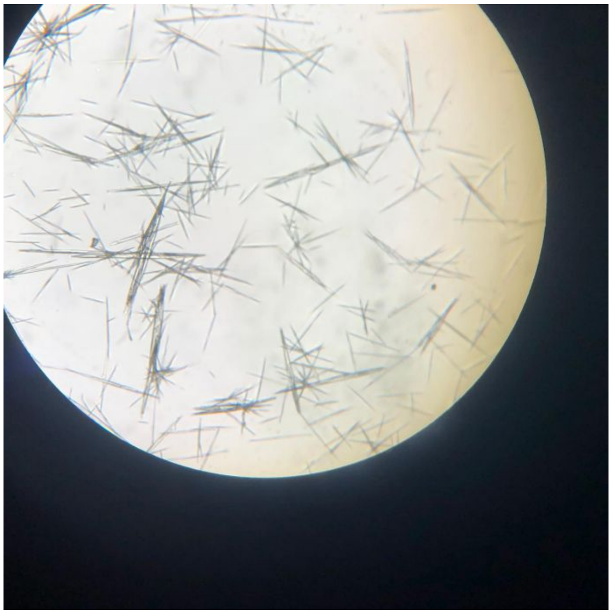

Figure S2. Optical image of Mg-TTA showing microcrystals of needle-like habit.

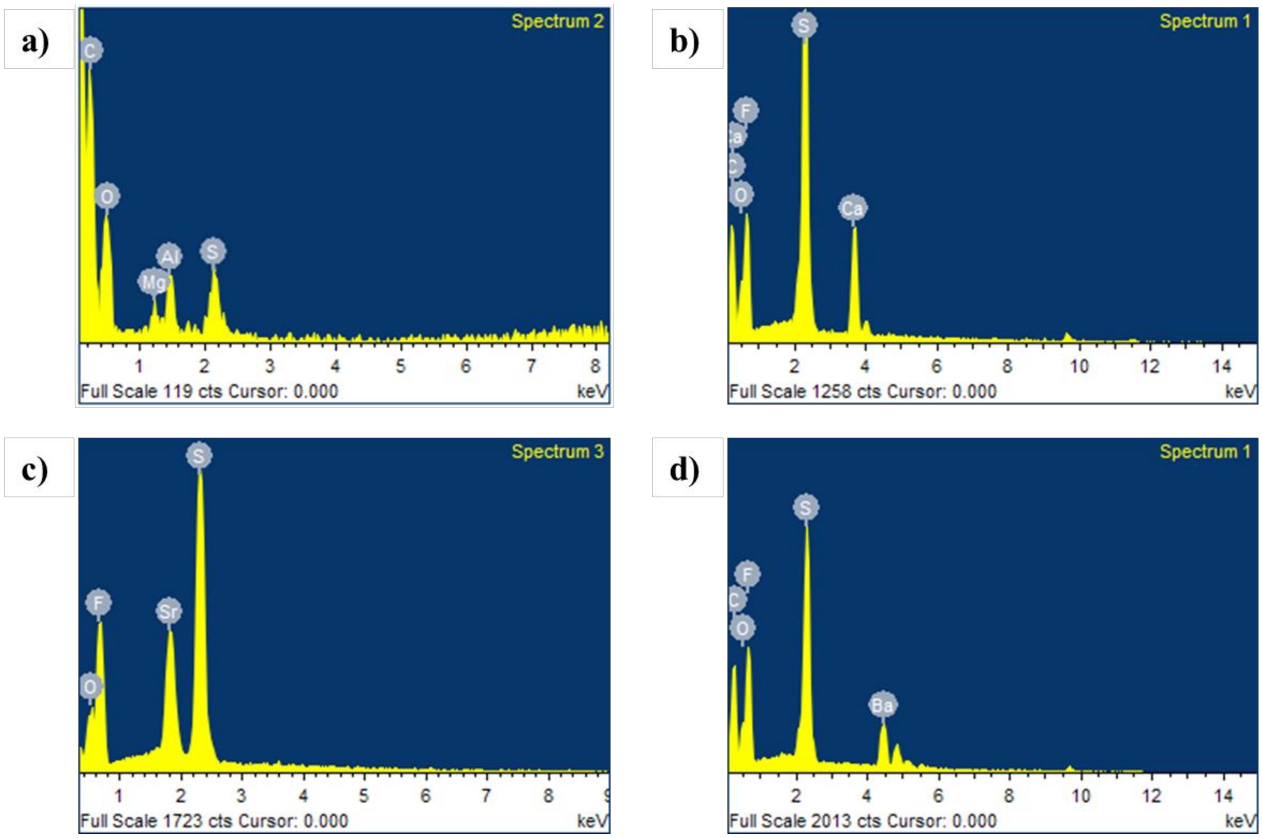

Figure S3. EDS analyses of Ae-TTA complexes: **a)** Mg-TTA, **b)** Ca-TTA, **c)** Sr-TTA, and **d)** Ba-TTA.

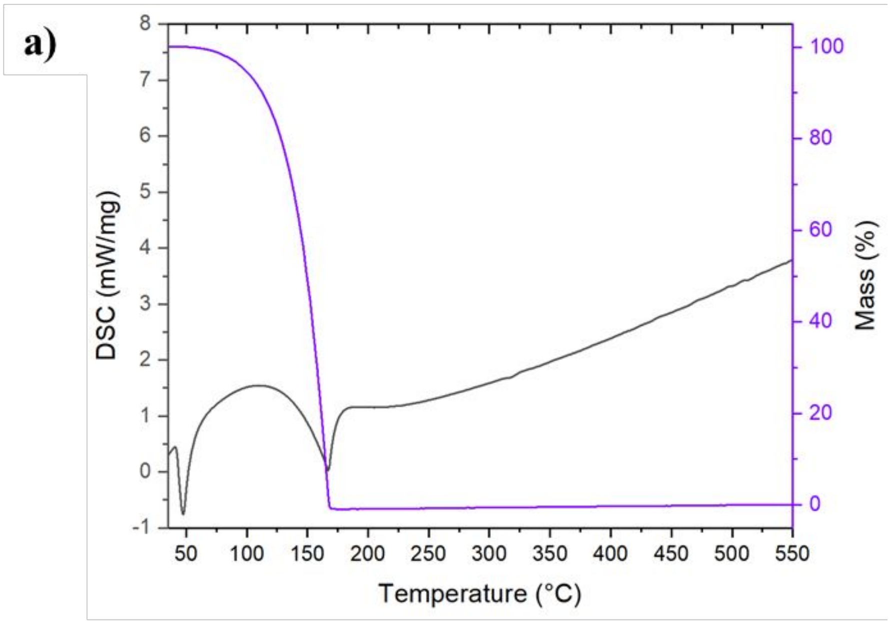

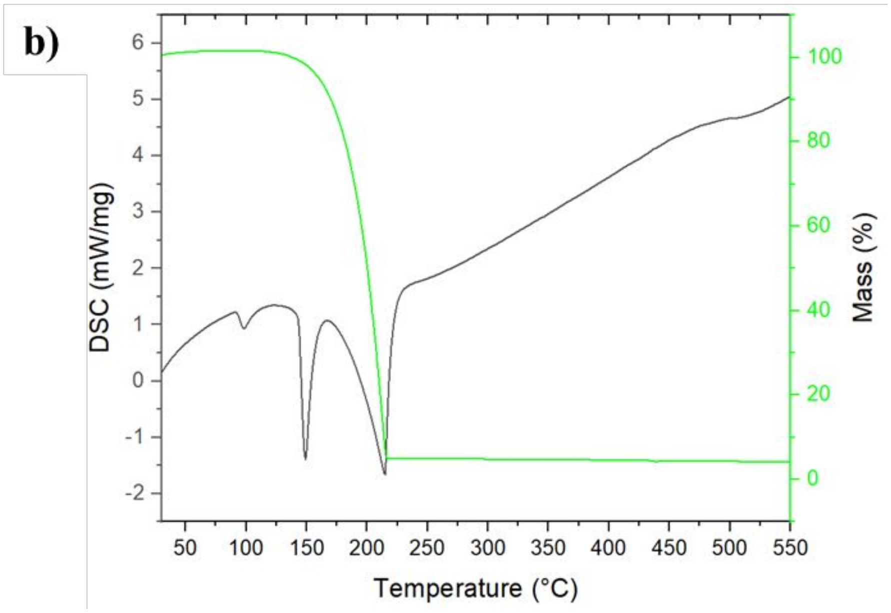

Figure S4. TGA-DSC profiles of: **a)** TTA and **b)** 2-MeIm.

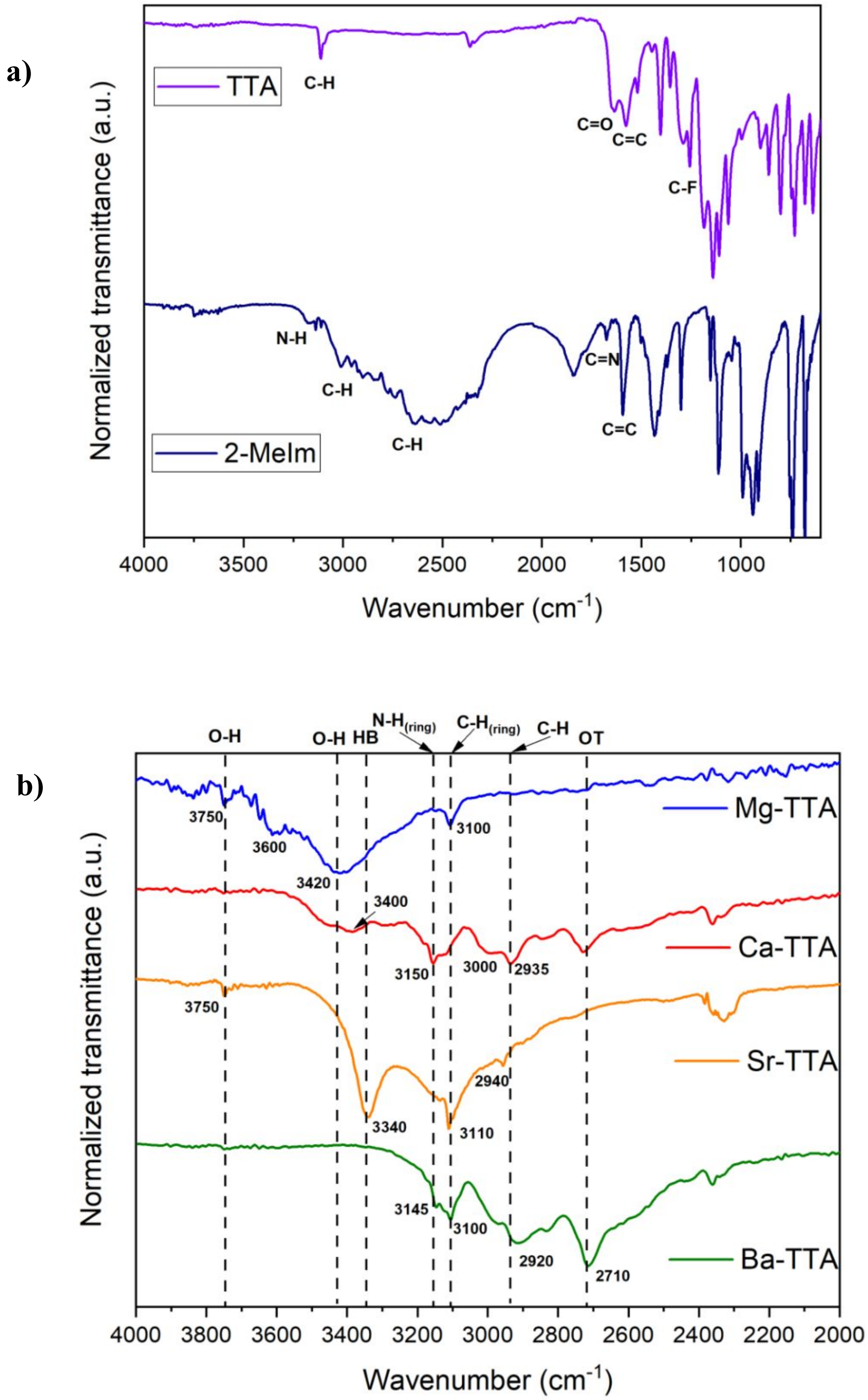

Figure S5. FTIR spectra of: **a)** free ligands TTA and 2-MeIm and **b)** Ae-complexes (4000 – 2000 cm^{-1})

Table S3. Information summarized from infrared analysis of Ae-TTA complexes **[74-79]**.

Vibrational Mode	TTA (free)	2-MeIm (free)	Mg–TTA	Ca–TTA	Sr–TTA	Ba–TTA
N–H stretch (imidazole ring)	—	3140–3100	3140–3100 (br)**	3140–3100 (br)**	3140–3100 (br)**	3140–3100 (br)**
C–H stretch (aromatic + aliphatic)	3100 (arom)	3140–3100 (arom), 2950–2870 (CH_3)	3050 (arom), 2920 (CH_3)	3050 (arom), 2920 (CH_3)	3050 (arom), 2920 (CH_3)	3050 (arom), 2920 (CH_3)
β-Diketone ν(C=O) (enolate)	1600	—	1610	1610	1636 (sh)	1606
β-Diketone ν(C=C) (conjugated enol)	1556	—	1582	1582	1578	1579
Imidazole ring ν(C=N)	—	1550*	1541	1541	1530	1539
C–F stretches (CF_3 group)	1290, 1257, 1185, 1108	—	1309, 1234, 1190, 1124	1292, 1230, 1184, 1119	1281, 1229, 1185, 1114	1283, 1230, 1183, 1122
Imidazole ring CH wag (out-of-plane)	—	905, 754, 677	860, 723, 650	860, 715, 644	860, 740, 635	860, 720, 640

**Free 2-MeIm exhibits multiple overlapping ring modes in the 1600–1500 cm⁻¹ region, so 1550 cm⁻¹ is an approximate representative value for the strongest C=N and C=C vibration.*
*** The N–H stretch of coordinated or protonated 2-MeIm in the complexes overlaps with aromatic C–H bands around 3140–3100 cm⁻¹, and no separate distinct N–H band was noted.*
